# Supplementary material for: LinDA: linear models for differential abundance analysis of microbiome compositional data
Source: Genome Biol. 2022 Apr 14;23:95. doi: 10.1186/s13059-022-02655-5 (PMC9012043; doi:10.1186/s13059-022-02655-5)
Supplement: Supplementary file 1 — Additional file 1 Supplementary notes. Table S1 lists some robust normalization methods [11–14]. Lemmas S1 – S4 present intermediate results for proving Theorem 1. The proof depends on some useful results from [50–53]. [file 13059_2022_2655_MOESM1_ESM.pdf]

Supplementary notes for "LinDA: linear models for differential abundance analysis of microbiome compositional data"

S1 Normalization approaches

Table S1: Some robust normalization methods

Method	Description
Trimmed mean of M-values (TMM) [11, Robinson & Oshlack, 2010]	TMM (in log scale) is the weighted mean of the log-ratio between the relative abundances and a referenced relative abundance after excluding the most abundant taxa and the taxa with the largest log-fold changes.
DESeq normalization (RLE) [12, Anders & Huber, 2010]	In RLE, the normalizing factor is the median of the ratios between the counts and the geometric mean of the counts of all samples.
Cumulative-sum scaling (CSS) [13, Paulson et al., 2013]	In CSS, counts are divided by the cumulative sum of counts, up to a quantile determined by a data-driven approach.
Geometric mean of pairwise ratios (GMPR) [14, Chen et al., 2018]	GMPR is the geometric mean of the medians of the ratios between the pairs of counts of two samples, which reverses the order of the two steps in the RLE.

Note that the first number in the square brackets represents the reference number in the manuscript.

S2 Technical details

In the following, we use $F_X(\cdot)$ to denote the cumulative distribution function of a random variable X. Denote by $o_{\mathbb{P}_m}$ $(O_{\mathbb{P}_m})$, $o_{\mathbb{P}_n}$ $(O_{\mathbb{P}_n})$, and $o_{\mathbb{P}}$ $(O_{\mathbb{P}})$ the corresponding rates of

convergence as $m \to \infty$, $n \to \infty$, and $m, n \to \infty$ simultaneously, respectively. We first introduce some useful lemmas before proving Theorem 1.

Lemma S1. *Under Condition (i) in Theorem 1, we have*

$$|\hat{\rho} - \rho| = o_{\mathbb{P}_n}(1).$$

Lemma S2. *Under Conditions (i), (ii), (iii), (v), and (ix) in Theorem 1, we have*

$$\max_i |\hat{\sigma}_i^2 - \sigma_i^2| = o_{\mathbb{P}}(1).$$

Lemma S3. *Under Conditions (i)–(viii) in Theorem 1, we have*

$$\sqrt{n}(\tilde{\alpha} - \bar{\alpha}) = o_{\mathbb{P}}(1).$$

Lemma S4. *Suppose Conditions (i)–(ix) in Theorem 1 are satisfied. Let m_0 be the number of true null hypotheses and*

$$V_{m,n}(t) = \sum_{i:\alpha_i=0} \mathbb{I}\left(|\sqrt{n}\hat{\alpha}_i| / \sqrt{\hat{\rho}\hat{\sigma}_i^2} > t\right),$$
$$S_{m,n}(t) = \sum_{i=1}^{m} \mathbb{I}\left(|\sqrt{n}\hat{\alpha}_i| / \sqrt{\hat{\rho}\hat{\sigma}_i^2} > t\right),$$
$$S_{\infty,n}(t) = \mathbb{P}\left(\left|\mathcal{E} + \sqrt{n}\alpha_i / \sqrt{\rho\sigma_i^2}\right| > t\right).$$

Then for any $0 < t_0 < \infty$,

$$\sup_{0<t<t_0} \left|m^{-1}S_{m,n}(t) - S_{\infty,n}(t)\right| = o_{\mathbb{P}}(1) \text{ and } \sup_{0<t<t_0} \left|m_0^{-1}V_{m,n}(t) - 2F_{n-d-2}(-t)\right| = o_{\mathbb{P}}(1).$$

Proof of Lemma S1. From Condition (i), we know that each element of $\mathbb{E}(\mathbf{z}_s\mathbf{z}_s^\top)$ is finite and $\det\{\mathbb{E}(\mathbf{z}_s\mathbf{z}_s^\top)\} > C$. We have $\hat{\rho} = \det(\hat{\mathbf{B}})/\det(\hat{\mathbf{A}})$ and $\rho = \det(\mathbf{B})/\det(\mathbf{A})$, where $\mathbf{A} = \mathbb{E}(\mathbf{z}_s\mathbf{z}_s^\top)$, $\hat{\mathbf{A}} = n^{-1}\sum_{s=1}^{n} \mathbf{z}_s\mathbf{z}_s^\top$, and $\mathbf{B}$ and $\hat{\mathbf{B}}$ are the principal submatrices obtained

by deleting the first row and first column of $\mathbf{A}$ and $\hat{\mathbf{A}}$ respectively. Thus we have that $|\det(\hat{\mathbf{B}}) - \det(\mathbf{B})| = o_{\mathbb{P}}(1)$ and $|\det(\hat{\mathbf{A}}) - \det(\mathbf{A})| = o_{\mathbb{P}}(1)$ using the law of large numbers. The Slutsky's theorem thus implies that $|\hat{\rho} - \rho| = o_{\mathbb{P}_n}(1)$. □

Proof of Lemma S2. Throughout the proof, we shall assume that $\varepsilon_{is}/\sigma_i$ is C-sub-Gaussian, which is indeed slightly weaker than Condition (iii). For any $\lambda > 0$, we have

$$\mathbb{E}[e^{\lambda\varepsilon_{is}} \mid \sigma_i] = \mathbb{E}\left[e^{\lambda\sigma_i(\varepsilon_{is}/\sigma_i)} \mid \sigma_i\right] \le e^{\lambda^2\sigma_i^2 C^2/2},$$
$$\mathbb{E}[e^{\lambda\bar{\varepsilon}_{is}} \mid \{\sigma_i\}] = \mathbb{E}\left[e^{\lambda\left\{(m-1)m^{-1}\varepsilon_{is} - m^{-1}\sum_{j\neq i}\varepsilon_{js}\right\}} \mid \{\sigma_i\}\right] \le e^{\lambda^2(\max_i \sigma_i^2)C^2/2}.$$

Thus $\bar{\varepsilon}_{is}$ conditional on $\{\sigma_i\}$ is sub-Gaussian by Condition (ii). Let $\bar{\boldsymbol{\theta}}_i = (\bar{\alpha}_i, \bar{\boldsymbol{\beta}}_i^\top)^\top$ and $\tilde{\boldsymbol{\theta}}_i = (\tilde{\alpha}_i, \tilde{\boldsymbol{\beta}}_i^\top)^\top$. Note that

$$\begin{aligned}
\tilde{\boldsymbol{\theta}}_i &= \bar{\boldsymbol{\theta}}_i + \left(\sum_{s=1}^n \mathbf{z}_s\mathbf{z}_s^\top\right)^{-1}\left(\sum_{s=1}^n \mathbf{z}_s\bar{\varepsilon}_{is}\right), \\
\hat{\sigma}_i^2 &= \frac{1}{n-d-2}\sum_{s=1}^n\left(W_{is} - \mathbf{z}_s^\top\tilde{\boldsymbol{\theta}}_i\right)^2 = \frac{1}{n-d-2}\sum_{s=1}^n\left(W_{is} - \mathbf{z}_s^\top\bar{\boldsymbol{\theta}}_i + \mathbf{z}_s^\top\bar{\boldsymbol{\theta}}_i - \mathbf{z}_s^\top\tilde{\boldsymbol{\theta}}_i\right)^2 \\
&= \frac{1}{n-d-2}\sum_{s=1}^n \bar{\varepsilon}_{is}^2 + \frac{2}{n-d-2}(\bar{\boldsymbol{\theta}}_i - \tilde{\boldsymbol{\theta}}_i)^\top\sum_{s=1}^n \mathbf{z}_s\bar{\varepsilon}_{is} \\
&\qquad + \frac{1}{n-d-2}(\bar{\boldsymbol{\theta}}_i - \tilde{\boldsymbol{\theta}}_i)^\top\left(\sum_{s=1}^n \mathbf{z}_s\mathbf{z}_s^\top\right)(\bar{\boldsymbol{\theta}}_i - \tilde{\boldsymbol{\theta}}_i) \\
&= \frac{1}{n-d-2}\sum_{s=1}^n \bar{\varepsilon}_{is}^2 - \frac{1}{n-d-2}\left(\sum_{s=1}^n \mathbf{z}_s\bar{\varepsilon}_{is}\right)^\top\left(\sum_{s=1}^n \mathbf{z}_s\mathbf{z}_s^\top\right)^{-1}\left(\sum_{s=1}^n \mathbf{z}_s\bar{\varepsilon}_{is}\right),
\end{aligned}$$

and for any $\delta > 0$,

$$\begin{aligned}
\mathbb{P}\left(\left|\hat{\sigma}_i^2 - \bar{\sigma}_i^2\right| > \delta\right) \le{}& \mathbb{P}\left(\left|\frac{1}{n-d-2}\sum_{s=1}^n \bar{\varepsilon}_{is}^2 - \bar{\sigma}_i^2\right| > \frac{\delta}{2}\right) \\
&+ \mathbb{P}\left\{\left(\sum_{s=1}^n \mathbf{z}_s\bar{\varepsilon}_{is}\right)^\top\left(\sum_{s=1}^n \mathbf{z}_s\mathbf{z}_s^\top\right)^{-1}\left(\sum_{s=1}^n \mathbf{z}_s\bar{\varepsilon}_{is}\right) > \frac{(n-d-2)\delta}{2}\right\}.
\end{aligned}$$

For the first term, we have

$$\mathbb{P}\left(\left|\frac{1}{n-d-2}\sum_{s=1}^{n}\bar{\varepsilon}_{is}^2-\bar{\sigma}_i^2\right|>\frac{\delta}{2}\right)\le\mathbb{P}\left\{\left|\frac{1}{n}\sum_{s=1}^{n}\bar{\varepsilon}_{is}^2-\bar{\sigma}_i^2\right|>\frac{(n-d-2)\delta}{4n}\right\}$$
$$+\mathbb{P}\left(\frac{d+2}{n-d-2}\bar{\sigma}_i^2>\frac{\delta}{4}\right).$$

For the second term, it can be shown that

$$\mathbb{P}\left\{\left(\sum_{s=1}^{n}\mathbf{z}_s\bar{\varepsilon}_{is}\right)^{\top}\left(\sum_{s=1}^{n}\mathbf{z}_s\mathbf{z}_s^{\top}\right)^{-1}\left(\sum_{s=1}^{n}\mathbf{z}_s\bar{\varepsilon}_{is}\right)>\frac{(n-d-2)\delta}{2}\right\}$$
$$\le\mathbb{P}\Bigg\{\left(\frac{1}{n}\sum_{s=1}^{n}\mathbf{z}_s\bar{\varepsilon}_{is}\right)^{\top}\left(\frac{1}{n}\sum_{s=1}^{n}\mathbf{z}_s\mathbf{z}_s^{\top}\right)^{-1}\left(\frac{1}{n}\sum_{s=1}^{n}\mathbf{z}_s\bar{\varepsilon}_{is}\right)>\frac{(n-d-2)\delta}{2n},$$
$$\left\|\frac{1}{n}\sum_{s=1}^{n}\mathbf{z}_s\mathbf{z}_s^{\top}-\mathbb{E}(\mathbf{z}_s\mathbf{z}_s^{\top})\right\|\le\delta_1\Bigg\}+\mathbb{P}\left\{\left\|\frac{1}{n}\sum_{s=1}^{n}\mathbf{z}_s\mathbf{z}_s^{\top}-\mathbb{E}(\mathbf{z}_s\mathbf{z}_s^{\top})\right\|>\delta_1\right\}$$
$$\le\mathbb{P}\left\{\left\|\frac{1}{n}\sum_{s=1}^{n}\mathbf{z}_s\bar{\varepsilon}_{is}\right\|>\sqrt{\frac{C(n-d-2)\delta}{n}}\right\}+\mathbb{P}\left\{\left\|\frac{1}{n}\sum_{s=1}^{n}\mathbf{z}_s\mathbf{z}_s^{\top}-\mathbb{E}(\mathbf{z}_s\mathbf{z}_s^{\top})\right\|>\delta_1\right\},$$

with $\delta_1>0$ being a small enough constant. In the above, the last inequality is due to the condition $\sigma_{\min}\{\mathbb{E}(\mathbf{z}_s\mathbf{z}_s^{\top})\}>C$ and Lemma S8 of [50, Zhou et al., 2021]. We conclude that $|\hat{\sigma}_i^2-\bar{\sigma}_i^2|$ has an exponential tail of the order $O(e^{-C_1 n})$ by using the Chernoff bound and the fact that the product of two sub-Gaussian variables is sub-exponential [51, Vershynin, 2018]. Thus by the union bound and Condition (ix), we have $\max_i|\hat{\sigma}_i^2-\bar{\sigma}_i^2|=o_{\mathbb{P}}(1)$. Observing that

$$|\bar{\sigma}_i^2-\sigma_i^2|=\left|\frac{1}{m}\left\{(m-2)\sigma_i^2+m^{-1}\sum_{i=1}^{m}\sigma_i^2\right\}-\sigma_i^2\right|=\left|\frac{-2}{m}\sigma_i^2-\frac{1}{m^2}\sum_{i=1}^{m}\sigma_i^2\right|=o_{\mathbb{P}_m}(1),$$

we obtain the desired result that $\max_i|\hat{\sigma}_i^2-\sigma_i^2|=o_{\mathbb{P}}(1)$. □

Proof of Lemma S3. We have

$$\sqrt{n}\tilde{\alpha}_i=\sqrt{n}\bar{\alpha}_i+\sqrt{n}\hat{\boldsymbol{\eta}}^{\top}n^{-1}\sum_{s=1}^{n}\mathbf{z}_s\bar{\varepsilon}_{is}=\sqrt{n}\alpha_i-\sqrt{n}\bar{\alpha}+U_i-U,$$

where

$$U_i = \hat{\boldsymbol{\eta}}^\top \frac{1}{\sqrt{n}} \sum_{s=1}^n \mathbf{z}_s \varepsilon_{is}, \quad U = \hat{\boldsymbol{\eta}}^\top \frac{1}{\sqrt{n}} \sum_{s=1}^n \mathbf{z}_s \left(\frac{1}{m} \sum_{i=1}^m \varepsilon_{is} \right),$$

and $\hat{\boldsymbol{\eta}}$ is the first row of $(n^{-1} \sum_{s=1}^n \mathbf{z}_s \mathbf{z}_s^\top)^{-1}$. We first prove that $U = o_{\mathbb{P}}(1)$. Using similar arguments as in the proof of Lemma S1, we have $|\hat{\boldsymbol{\eta}} - \boldsymbol{\eta}| = o_{\mathbb{P}_n}(1)$, where $\boldsymbol{\eta}$ is the first row of $\{\mathbb{E}(\mathbf{z}_s \mathbf{z}_s^\top)\}^{-1}$. Under Conditions (i), (iii), and (v), $\mathbf{z}_s(\sum_{i=1}^m \varepsilon_{is})/\sqrt{m}$ are conditionally i.i.d. given $\sigma_1, \ldots, \sigma_m$. Thus,

$$\mathbb{E}\left\{ \mathbf{z}_s \left(\frac{1}{\sqrt{m}} \sum_{i=1}^m \varepsilon_{is} \right) \;\middle|\; \sigma_1, ..., \sigma_m \right\} = 0,$$

$$\mathbb{E}\left\{ (\mathbf{z}_s \odot \mathbf{z}_s) \left(\frac{1}{\sqrt{m}} \sum_{i=1}^m \varepsilon_{is} \right)^2 \;\middle|\; \sigma_1, \ldots, \sigma_m \right\} = \frac{\mathbb{E}(\mathbf{z}_s \odot \mathbf{z}_s)}{m} \sum_{i=1}^m \sigma_i^2,$$

where $\odot$ denotes the Hadamard product (element-wise product). The above implies that

$$\frac{1}{\sqrt{n}} \sum_{s=1}^n \mathbf{z}_s \left(\frac{1}{\sqrt{m}} \sum_{i=1}^m \varepsilon_{is} \right) = O_{\mathbb{P}_n}(1)$$

whenever $\sum_{i=1}^m \sigma_i^2/m < \infty$. Using Condition (ii), we have $\mathbb{P}(\sum_{i=1}^m \sigma_i^2/m < \infty) = 1$. Thus $U = O_{\mathbb{P}}(m^{-1/2})$. Recall that

$$\widehat{\mathrm{mode}}(\{X_i\}_{i=1}^m) = \arg\max_{x \in \mathbb{R}} \frac{1}{mh} \sum_{i=1}^m K\left(\frac{x - X_i}{h} \right).$$

It is not hard to see that $\widehat{\mathrm{mode}}(\{X_i + a\}_{i=1}^m) = \widehat{\mathrm{mode}}(\{X_i\}_{i=1}^m) + a$, for any a, which may be related to m but is independent of i. We then have

$$\widehat{\mathrm{mode}}(\{\sqrt{n}\tilde{\alpha}_i\}_{i=1}^m) = \widehat{\mathrm{mode}}(\{\sqrt{n}\alpha_i - \sqrt{n}\bar{\alpha} + U_i - U\}_{i=1}^m) = \widehat{\mathrm{mode}}(\{\sqrt{n}\alpha_i + U_i\}_{i=1}^m) - \sqrt{n}\bar{\alpha} - U.$$

Therefore, we only need to show that $\tilde{M} := \widehat{\mathrm{mode}}(\{\sqrt{n}\alpha_i + U_i\}_{i=1}^m) = o_{\mathbb{P}}(1)$. To this end, let

$$f_{m,h}(x) = \frac{1}{mh} \sum_{i=1}^m K\left(\frac{x - (\sqrt{n}\alpha_i + U_i)}{h} \right).$$

Given Condition (vi), we have that for large enough n,

$$\begin{aligned}|f_n(\tilde{M};\rho)-f_n(0;\rho)| &\le |f_n(\tilde{M};\rho)-f_{m,h}(\tilde{M})|+|f_{m,h}(\tilde{M})-f_n(0;\rho)|\\ &= |f_n(\tilde{M};\rho)-f_{m,h}(\tilde{M})|+\left|\sup_{x\in\mathbb{R}} f_{m,h}(x)-\sup_{x\in\mathbb{R}} f_n(x;\rho)\right|\\ &\le 2\sup_{x\in\mathbb{R}}|f_{m,h}(x)-f_n(x;\rho)|,\end{aligned}$$

and then it boils down to show that

$$\sup_{x\in\mathbb{R}}|f_{m,h}(x)-f_n(x;\rho)| = o_{\mathbb{P}}(1).$$

Note that

$$f_n(x;a)=\int\int \frac{1}{\sqrt{a}u}\phi\left(\frac{x-v}{\sqrt{a}u}\right)dF_{\sigma_i}(u)dF_{\sqrt{n}\alpha_i}(v)$$

for any $a>0$, where $\phi(\cdot)$ denotes the density function of the standard normal distribution. It implies that $f_n(x;a)$ is uniformly continuous and bounded uniformly over n and $a>C$. In other words, for any $\epsilon>0$, there exists a $\delta>0$ such that $\sup_{n,a>C,|x_1-x_2|<\delta}|f_n(x_1;a)-f_n(x_2;a)|<\epsilon$ and $\sup_{n,a>C,x\in\mathbb{R}} f_n(x;a)<\infty$. Besides, $\sup_{n,x\in\mathbb{R}}|f_n(x;\hat{\rho})-f_n(x;\rho)|$ can be made arbitrarily small as long as $|\hat{\rho}-\rho|$ is small enough and $\rho>C>0$. Thus we have

$$\begin{aligned}&\mathbb{P}\left\{\sup_{x\in\mathbb{R}}|f_{m,h}(x)-f_n(x;\rho)|>\delta\right\}\\ &\quad\le \mathbb{P}\left\{\sup_{x\in\mathbb{R}}|f_{m,h}(x)-f_n(x;\rho)|>\delta, |\hat{\rho}-\rho|\le\delta_1\right\}+\mathbb{P}\left(|\hat{\rho}-\rho|>\delta_1\right)\\ &\quad\le \mathbb{P}\left\{\sup_{x\in\mathbb{R}}|f_{m,h}(x)-f_n(x;\hat{\rho})|>\delta/2, |\hat{\rho}-\rho|\le\delta_1\right\}+\mathbb{P}\left(|\hat{\rho}-\rho|>\delta_1\right)\\ &\quad= \int_{|u-\rho|\le\delta_1}\mathbb{P}\left\{\sup_{x\in\mathbb{R}}|f_{m,h}(x)-f_n(x;\hat{\rho})|>\delta/2 \mid \hat{\rho}=u\right\}dF_{\hat{\rho}}(u)+\mathbb{P}\left(|\hat{\rho}-\rho|>\delta_1\right)\end{aligned}$$

for any $\delta>0$ and small enough $\delta_1>0$. Because $|\hat{\rho}-\rho|=o_{\mathbb{P}_n}(1)$ as shown in Lemma S1, our goal narrows down to proving that for any $\delta>0$ and $\epsilon>0$, there exists a $\xi>0$ such

that when m is large enough,

$$\sup_{n,\,|\hat{\rho}-\rho|\le\xi} \mathbb{P}\left\{\sup_{x\in\mathbb{R}} |f_{m,h}(x) - f_n(x;\hat{\rho})| > \delta \mid \hat{\rho}\right\} < \epsilon.$$

To show the above displayed inequality holds for some $\xi > 0$, it is sufficient to show

$$\sup_{\substack{n,\,x\in\mathbb{R},\\ |\hat{\rho}-\rho|\le\xi}} |\mathbb{E}\{f_{m,h}(x) \mid \hat{\rho}\} - f_n(x;\hat{\rho})| < \epsilon \tag{S1}$$

and

$$\sup_{n,\,|\hat{\rho}-\rho|\le\xi} \mathbb{E}\left[\sup_{x\in\mathbb{R}} |f_{m,h}(x) - \mathbb{E}\{f_{m,h}(x) \mid \hat{\rho}\}|^2 \mid \hat{\rho}\right] < \epsilon \tag{S2}$$

are fulfilled for some small enough $\xi > 0$.

For (S1), using $\int_{-\infty}^{\infty} K(y)dy = 1$ with $K(y) \ge 0$, we observe that

$$\begin{aligned}
&\sup_{\substack{n,\,x\in\mathbb{R},\\ |\hat{\rho}-\rho|\le\xi}} |\mathbb{E}\{f_{m,h}(x) \mid \hat{\rho}\} - f_n(x;\hat{\rho})| \\
&\quad = \sup_{\substack{n,\,x\in\mathbb{R},\\ |\hat{\rho}-\rho|\le\xi}} \left|\int_{-\infty}^{\infty} \frac{1}{h} K\left(\frac{x-y}{h}\right) f_n(y;\hat{\rho})dy - f_n(x;\hat{\rho})\right| \\
&\quad = \sup_{\substack{n,\,x\in\mathbb{R},\\ |\hat{\rho}-\rho|\le\xi}} \left|\int_{-\infty}^{\infty} \frac{1}{h} K\left(\frac{y}{h}\right) \{f_n(x-y;\hat{\rho}) - f_n(x;\hat{\rho})\}\, dy\right| \\
&\quad = \sup_{\substack{n,\,x\in\mathbb{R},\\ |\hat{\rho}-\rho|\le\xi}} \left|\int_{|y|\le\nu} \frac{1}{h} K\left(\frac{y}{h}\right) \{f_n(x-y;\hat{\rho}) - f_n(x;\hat{\rho})\}\, dy\right| \\
&\qquad\quad + \sup_{\substack{n,\,x\in\mathbb{R},\\ |\hat{\rho}-\rho|\le\xi}} \left|\int_{|y|>\nu} \frac{1}{h} K\left(\frac{y}{h}\right) \{f_n(x-y;\hat{\rho}) - f_n(x;\hat{\rho})\}\, dy\right| \\
&\quad \le \sup_{\substack{n,\,|\hat{\rho}-\rho|\le\xi,\\ x\in\mathbb{R},\,|y|\le\nu}} |f_n(x-y;\hat{\rho}) - f_n(x;\hat{\rho})| \int_{|u|\le\nu/h} K(u)du \\
&\qquad\quad + \sup_{\substack{n,\,x\in\mathbb{R},\\ |\hat{\rho}-\rho|\le\xi}} f_n(x;\hat{\rho}) \int_{|u|>\nu/h} K(u)du.
\end{aligned} \tag{S3}$$

Due to the condition that $f_n(x; a)$ is uniformly continuous and upper bounded uniformly over n, (S3) is less than ϵ for some small enough $\xi > 0$ and $\nu > 0$ (depending on ϵ). It completes (S1) for some ξ.

For (S2), note that U_i's have the same distribution as $\sqrt{\hat{\rho}}\varepsilon_{is}$ and are independent given $\hat{\rho}$. Let $X_i = \sqrt{n}\alpha_i + U_i$. Define

$$\varphi_m(u) = m^{-1} \sum_{i=1}^{m} e^{\imath u X_i}.$$

The inverse Fourier transformation provides

$$K(y) = (2\pi)^{-1} \int_{-\infty}^{\infty} k(u) e^{\imath u y} du.$$

After plugging this expression into the definition of $f_{m,h}$, it shows that

$$\begin{aligned} f_{m,h}(x) &= \frac{1}{mh} \sum_{i=1}^{m} K\left(\frac{x - X_i}{h}\right) \\ &= (2\pi m h)^{-1} \sum_{i=1}^{m} \int_{-\infty}^{\infty} k(u) e^{\imath u \frac{x - X_i}{h}} du \\ &= (2\pi m)^{-1} \sum_{i=1}^{m} \int_{-\infty}^{\infty} k(hu) e^{\imath u (x - X_i)} du \\ &= (2\pi)^{-1} \int_{-\infty}^{\infty} e^{-\imath u x} k(hu) \varphi_m(u) du, \end{aligned}$$

where the last equality is because $k(u)$ is even. This result further implies

$$\sup_{x \in \mathbb{R}} |f_{m,h}(x) - \mathbb{E}\{f_{m,h}(x) \mid \hat{\rho}\}| \le (2\pi)^{-1} \int_{-\infty}^{\infty} |k(hu)| \, |\varphi_m(u) - \mathbb{E}\{\varphi_m(u) \mid \hat{\rho}\}| \, du.$$

Using the above inequality, the Cauchy-Schwartz inequality, and Euler's identity (i.e., $|e^{\imath x}| = 1$), it shows that the left hand side of (S2) satisfies

$$\sup_{n, \, |\hat{\rho} - \rho| \le \xi} \mathbb{E}\left[\sup_{x \in \mathbb{R}} |f_{m,h}(x) - \mathbb{E}\{f_{m,h}(x) \mid \hat{\rho}\}|^2 \mid \hat{\rho}\right]$$

$$
\begin{aligned}
&\leq \sup_{n,\,|\hat\rho-\rho|\leq\xi} \mathbb{E}\left(\left[(2\pi)^{-1}\int_{-\infty}^{\infty}|k(hu)|\,|\varphi_m(u)-\mathbb{E}\{\varphi_m(u)\mid\hat\rho\}|\,du\right]^2 \mid \hat\rho\right)\\
&\leq \sup_{n,\,|\hat\rho-\rho|\leq\xi} (2\pi)^{-2}\int_{-\infty}^{\infty}|k(hu)|du\int_{-\infty}^{\infty}|k(hu)|\,\mathbb{E}\left[|\varphi_m(u)-\mathbb{E}\{\varphi_m(u)\mid\hat\rho\}|^2\mid\hat\rho\right]du\\
&= \sup_{n,\,|\hat\rho-\rho|\leq\xi} (2\pi)^{-2}m^{-1}\int_{-\infty}^{\infty}|k(hu)|du\int_{-\infty}^{\infty}|k(hu)|\,\mathbb{E}\left[|e^{\imath uX_i}-\mathbb{E}\{e^{\imath uX_i}\mid\hat\rho\}|^2\mid\hat\rho\right]du\\
&\leq \pi^{-2}m^{-1}h^{-2}\left\{\int_{-\infty}^{\infty}|k(u)|du\right\}^2\to 0,
\end{aligned}
$$

where the result of converging to 0 is due to Conditions (vii) and (viii). Therefore, (S2) is satisfied, which completes the proof. □

Proof of Lemma S4. In the following, we focus on showing $\sup_{0<t<t_0}|m^{-1}S_{m,n}(t)-S_{\infty,n}(t)| = o_{\mathbb{P}}(1)$. The proof of the second statement can be obtained by similar arguments, and thus is omitted.

Let

$$S^-_{m,n}(t)=\sum_{i=1}^m \mathbb{I}\left(\sqrt{n}\hat\alpha_i/\sqrt{\hat\rho\hat\sigma_i^2}<-t\right).$$

The goal is to show

$$\sup_{0<t<t_0}\left|\frac{1}{m}S^-_{m,n}(t)-\mathbb{P}\left(\mathcal{E}+\sqrt{n}\alpha_i/\sqrt{\rho\sigma_i^2}<-t\right)\right|=o_{\mathbb{P}}(1).$$

Recall in the proof of Lemma S3, we have

$$\sqrt{n}\hat\alpha_i=\sqrt{n}(\tilde\alpha_i+\tilde\alpha)=\sqrt{n}\alpha_i+\sqrt{n}(\tilde\alpha-\bar\alpha)+U_i-U,$$

where $U_i/\sqrt{\hat\rho\sigma_i^2}\sim^{\text{i.i.d.}} N(0,1)$, $U=o_{\mathbb{P}}(1)$, and $\sqrt{n}(\tilde\alpha-\bar\alpha)=o_{\mathbb{P}}(1)$. These results imply that

$$\frac{1}{m}S^-_{m,n}(t)=\frac{1}{m}\sum_{i=1}^m\mathbb{I}\left\{\frac{U_i}{\sqrt{\hat\rho\sigma_i^2}}+\frac{\alpha_i}{\sqrt{\hat\rho\sigma_i^2/n}}<-t\frac{\hat\sigma_i}{\sigma_i}+\frac{U-\sqrt{n}(\tilde\alpha-\bar\alpha)}{\sqrt{\hat\rho\sigma_i^2}}\right\},$$

and

$$
\begin{aligned}
&\mathbb{P}\left\{\sup_{0<t<t_0}\left|\frac{1}{m}S^-_{m,n}(t)-\mathbb{P}\left(\mathcal{E}+\alpha_i/\sqrt{\rho\sigma_i^2/n}<-t\right)\right|>\delta\right\}\\
&\le \mathbb{P}\Bigg[\sup_{0<t<t_0}\left|\frac{1}{m}\sum_{i=1}^m \mathbb{I}\left\{\frac{U_i}{\sqrt{\hat\rho\sigma_i^2}}+\frac{\alpha_i}{\sqrt{\hat\rho\sigma_i^2/n}}<-t\frac{\hat\sigma_i}{\sigma_i}+\frac{U-\sqrt{n}(\tilde\alpha-\bar\alpha)}{\sqrt{\hat\rho\sigma_i^2}}\right\}\right.\\
&\qquad\qquad \left.-\mathbb{P}\left(\mathcal{E}+\frac{\alpha_i}{\sqrt{\rho\sigma_i^2/n}}<-t\right)\right|>\delta,\ \sup_i\left|\frac{\hat\sigma_i}{\sigma_i}-1\right|\le\delta_1,\ \sup_i\left|\frac{U-\sqrt{n}(\tilde\alpha-\bar\alpha)}{\sqrt{\hat\rho\sigma_i^2}}\right|\le\delta_2\Bigg]\\
&\quad+\mathbb{P}\left(\sup_i\left|\frac{\hat\sigma_i}{\sigma_i}-1\right|>\delta_1\right)+\mathbb{P}\left\{\sup_i\left|\frac{U-\sqrt{n}(\tilde\alpha-\bar\alpha)}{\sqrt{\hat\rho\sigma_i^2}}\right|>\delta_2\right\}\\
&\le \mathbb{P}\left\{\sup_{0<t<t_0}\left|\frac{1}{m}\sum_{i=1}^m \mathbb{I}\left(\frac{U_i}{\sqrt{\hat\rho\sigma_i^2}}+\frac{\alpha_i}{\sqrt{\hat\rho\sigma_i^2/n}}<-t-t\delta_1-\delta_2\right)-\mathbb{P}\left(\mathcal{E}+\frac{\alpha_i}{\sqrt{\rho\sigma_i^2/n}}<-t\right)\right|>\delta\right\}\\
&\quad+\mathbb{P}\left\{\sup_{0<t<t_0}\left|\frac{1}{m}\sum_{i=1}^m \mathbb{I}\left(\frac{U_i}{\sqrt{\hat\rho\sigma_i^2}}+\frac{\alpha_i}{\sqrt{\hat\rho\sigma_i^2/n}}<-t+t\delta_1+\delta_2\right)-\mathbb{P}\left(\mathcal{E}+\frac{\alpha_i}{\sqrt{\rho\sigma_i^2/n}}<-t\right)\right|>\delta\right\}\\
&\quad+o(1)
\end{aligned}
$$

for any positive constants δ, δ_1, and δ_2, where the last step is due to $\rho > C$, $\sigma_i > C$, and the results from Lemmas S1–S3. Thus we only need to show that for any $\delta > 0$ and $\epsilon > 0$, there exist $\xi > 0$, $\delta_1 \neq 0$ and $\delta_2 \neq 0$ such that for large enough m,

$$
\begin{aligned}
\sup_{n,\,|\hat\rho-\rho|<\xi}\mathbb{P}\Bigg\{\sup_{0<t<t_0}\Bigg|\frac{1}{m}\sum_{i=1}^m \mathbb{I}\left(\frac{U_i}{\sqrt{\hat\rho\sigma_i^2}}+\frac{\alpha_i}{\sqrt{\hat\rho\sigma_i^2/n}}<-t+t\delta_1+\delta_2\right)&\\
-\mathbb{P}\left(\mathcal{E}+\frac{\alpha_i}{\sqrt{\rho\sigma_i^2/n}}<-t\right)\Bigg|>\delta\ \Bigg|\ \hat\rho\Bigg\}&<\epsilon,
\end{aligned}
$$

or sufficiently,

$$\sup_{n,\,|\hat\rho-\rho|<\xi} \mathbb{P}\Bigg\{ \sup_{0<t<t_0} \Bigg| \frac{1}{m}\sum_{i=1}^{m} \mathbb{I}\Bigg(\frac{U_i}{\sqrt{\hat\rho\sigma_i^2}} + \frac{\alpha_i}{\sqrt{\hat\rho\sigma_i^2/n}} < -t + t\delta_1 + \delta_2 \Bigg) - \mathbb{P}\Bigg(\mathcal{E} + \frac{\alpha_i}{\sqrt{\hat\rho\sigma_i^2/n}} < -t + t\delta_1 + \delta_2 \,\Bigg|\, \hat\rho \Bigg) \Bigg| > \delta \,\Bigg|\, \hat\rho \Bigg\} < \epsilon, \tag{S4}$$

$$\sup_{\substack{n,\,|\hat\rho-\rho|<\xi,\\ 0<t<t_0}} \Bigg| \mathbb{P}\Bigg(\mathcal{E} + \frac{\alpha_i}{\sqrt{\hat\rho\sigma_i^2/n}} < -t + t\delta_1 + \delta_2 \,\Bigg|\, \hat\rho \Bigg) - \mathbb{P}\Bigg(\mathcal{E} + \frac{\alpha_i}{\sqrt{\hat\rho\sigma_i^2/n}} < -t \,\Bigg|\, \hat\rho \Bigg) \Bigg| < \epsilon, \tag{S5}$$

and

$$\sup_{\substack{n,\,|\hat\rho-\rho|<\xi,\\ 0<t<t_0}} \Bigg| \mathbb{P}\Bigg(\mathcal{E} + \frac{\alpha_i}{\sqrt{\hat\rho\sigma_i^2/n}} < -t \,\Bigg|\, \hat\rho \Bigg) - \mathbb{P}\Bigg(\mathcal{E} + \frac{\alpha_i}{\sqrt{\rho\sigma_i^2/n}} < -t \Bigg) \Bigg| < \epsilon. \tag{S6}$$

First, (S4) is a direct result of applying the Glivenko-Cantelli theorem [52, Wainwright, 2019]. For (S5), we note that the cumulative distribution function of $\mathcal{E} + \alpha_i/\sqrt{a\sigma_i^2/n}$ for any $a > 0$, denoted by $G_n(\cdot; a)$, can be expressed as

$$G_n(x; a) = \int_{-\infty}^{\infty} \Phi\left(x - u\right) dF_{\alpha_i/\sqrt{a\sigma_i^2/n}}(u) = \int_{-\infty}^{\infty} \Phi\left(x - \sqrt{\frac{\rho}{a}}u\right) dF_{\alpha_i/\sqrt{\rho\sigma_i^2/n}}(u),$$

where $\Phi(\cdot)$ represents the cumulative distribution function of the standard normal distribution. Thus $G_n(x; a)$ is equicontinuous uniformly over n and $a > 0$. In other words, for any $\epsilon > 0$, there exists a $\delta > 0$ such that

$$\sup_{\substack{n,\,a>0,\\ |x_1-x_2|<\delta}} |G_n(x_1; a) - G_n(x_2; a)| < \epsilon,$$

which verifies the (S5). Further,

$$\sup_{\substack{n,\,|\hat\rho-\rho|<\xi,\\ |x|<t_0}} |G_n(x; \hat\rho) - G_n(x; \rho)|$$

can be arbitrarily small as long as ξ is small enough, which confirms (S6). □

Proof of Theorem 1. Observe that

$$\left|\widehat{\mathrm{FDP}}(t) - \frac{2F_{n-d-2}(-t)}{S_{\infty,n}(t)}\right| = \left|2F_{n-d-2}(-t)\left\{\frac{1}{S_{m,n}(t)/m} - \frac{1}{S_{\infty,n}(t)}\right\}\right|,$$

where $S_{m,n}(t)$ and $S_{\infty,n}(t)$ are defined in Lemma S4. Together with Lemma S4 and Condition (x), we deduce that there exists some t_0 such that $t^* < t_0$ for large enough n,

$$\begin{aligned}
&\sup_{0<t<t_0}\left|\frac{V_{m,n}(t)}{1\vee S_{m,n}(t)} - \frac{m_0}{m}\frac{2F_{n-d-2}(-t)}{S_{\infty,n}(t)}\right| \\
=&\sup_{0<t<t_0}\left|\frac{V_{m,n}(t)}{1\vee S_{m,n}(t)} - \frac{2F_{n-d-2}(-t)}{\{1\vee S_{m,n}(t)\}/m_0} + \frac{2F_{n-d-2}(-t)}{\{1\vee S_{m,n}(t)\}/m_0} - \frac{m_0}{m}\frac{2F_{n-d-2}(-t)}{S_{\infty,n}(t)}\right| \\
\le&\sup_{0<t<t_0}\left|\frac{m_0^{-1}V_{m,n}(t) - 2F_{n-d-2}(-t)}{\{1\vee S_{m,n}(t)\}/m_0}\right| + \sup_{0<t<t_0}\left|\frac{2m_0F_{n-d-2}(-t)}{m}\left[\frac{1}{\{1\vee S_{m,n}(t)\}/m} - \frac{1}{S_{\infty,n}(t)}\right]\right| \\
=&o_{\mathbb{P}}(1),
\end{aligned}$$

and

$$\sup_{0<t<t_0}\left|\widehat{\mathrm{FDP}}(t) - \frac{2F_{n-d-2}(-t)}{S_{\infty,n}(t)}\right| = \sup_{0<t<t_0}\left|2F_{n-d-2}(-t)\left\{\frac{1}{S_{m,n}(t)/m} - \frac{1}{S_{\infty,n}(t)}\right\}\right| = o_{\mathbb{P}}(1).$$

Therefore, we have

$$\begin{aligned}
\frac{V_{m,n}(t^*)}{1\vee S_{m,n}(t^*)} &\le \frac{V_{m,n}(t^*)}{1\vee S_{m,n}(t^*)} - \frac{m_0}{m}\frac{2F_{n-d-2}(-t^*)}{S_{\infty,n}(t^*)} + \frac{2F_{n-d-2}(-t^*)}{S_{\infty,n}(t^*)} - \widehat{\mathrm{FDP}}(t^*) + \widehat{\mathrm{FDP}}(t^*) \\
&\le q + o_{\mathbb{P}}(1).
\end{aligned}$$

The conclusion follows by using Lemma 8.3 of [53, Cao et al., 2021]. □
