# Supplementary material for: LinDA: linear models for differential abundance analysis of microbiome compositional data
Source: Genome Biol. 2022 Apr 14;23:95. doi: 10.1186/s13059-022-02655-5 (PMC9012043; doi:10.1186/s13059-022-02655-5)
Supplement: Supplementary file 2 — Additional file 2 Supplementary figures. Fig. S1 and S2 compare the proposed method LinDA with different zero-handling approaches under settings S6C0 and S0C0. Fig. S3 depicts the results of LinDA, CLR-OLS and MaAsLin2 with different normalization approaches under setting S0C0. Fig. S4–S10 and S12–S14 show the results of settings S0C1, S0C2, S1C0, S2C0, S4C0, S5C0, S6C0, S7C0, S8.1C0, and S8.2C0, respectively. The comparison between disabling and enabling zero treatment of the ANCOM-BC method is depicted in Fig. S11 under setting S6C0. Fig. S15 shows the results of setting S0C0 with stronger compositional effects. Fig. S16–S19 show the effect size plots and volcano plots for the four datasets (CDI, IBD, RA, and SMOKE) respectively. Fig. S20–S30 present the full result of all methods under different simulation settings. [file 13059_2022_2655_MOESM2_ESM.pdf]

Supplementary figures for “LinDA: linear models for differential abundance analysis of microbiome compositional data”

S1 Additional main comparisons of numerical studies

Fig. S1 and Fig. S2 compare the proposed method LinDA with different zero-handling approaches under settings S6C0 and S0C0. Fig. S3 depicts the results of LinDA, CLR-OLS and MaAsLin2 with different normalization approaches under setting S0C0. Fig. S4–S10, S12 and S13–S14 show the results of settings S0C1, S0C2, S1C0, S2C0, S4C0, S5C0, S6C0, S7C0, S8.1C0, and S8.2C0, respectively. The comparison between disabling and enabling zero treatment of the ANCOM-BC method is depicted in Fig. S11 under setting S6C0. Fig. S15 shows the results of setting S0C0 with stronger compositional effects.

S2 Additional results of real data applications

Fig. S16–S19 show the effect size plots and volcano plots for the four datasets (CDI, IBD, RA, and SMOKE) respectively.

S3 Full comparisons of numerical studies

Fig. S20–S30 present the full result of all methods under different simulation settings.

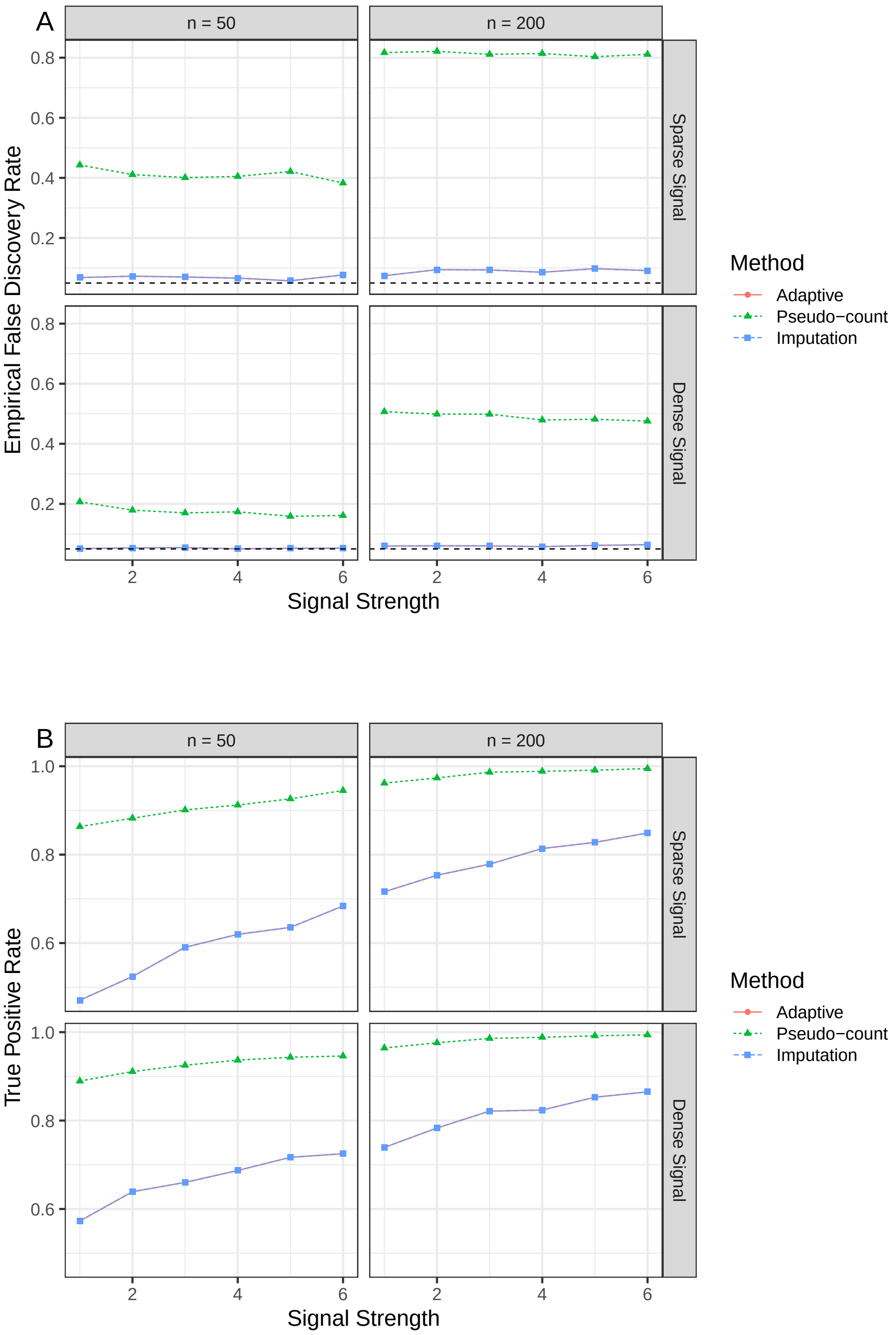

Fig. S1: Performance of LinDA with different zero-handling approaches (S6C0: 10-fold difference in library size, a binary covariate). Empirical false discovery rate (A) and true positive rates (B) were averaged over 100 simulation runs. The dashed horizontal line (A) indicates the target FDR level of 0.05. Note that the red and blue lines are overlapped as the covariate and sequencing depth are significantly correlated.

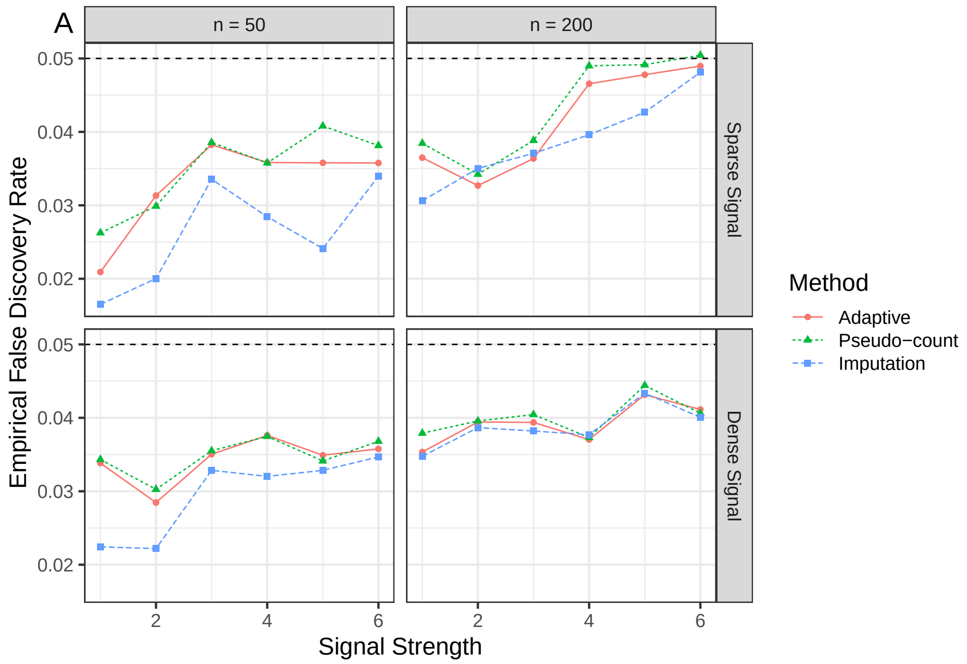

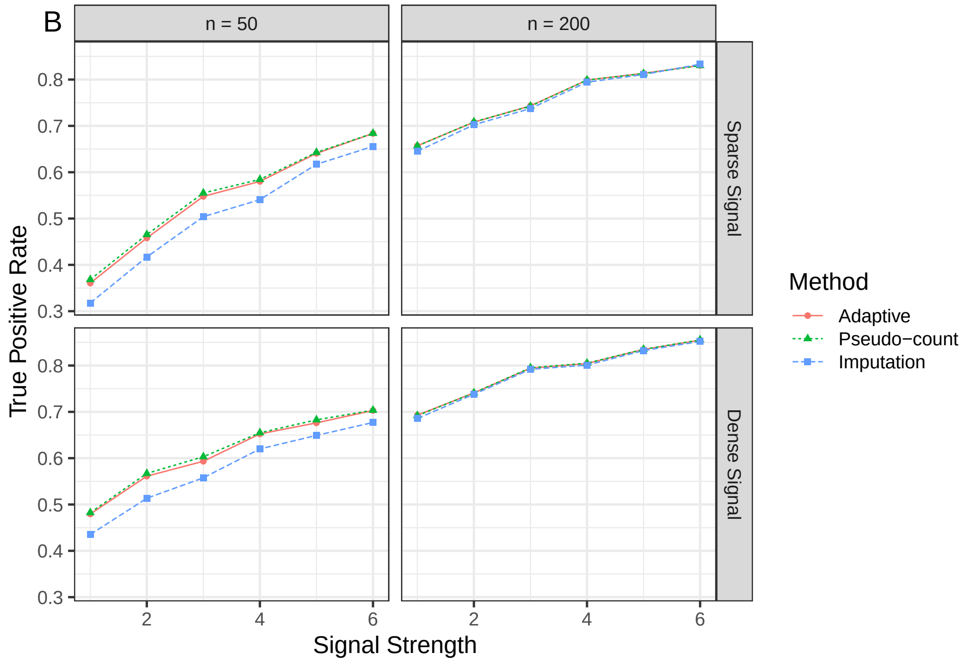

Fig. S2: Performance of LinDA with different zero-handling approaches (S0C0: log normal abundance distribution, a binary covariate). Empirical false discovery rate (A) and true positive rates (B) were averaged over 100 simulation runs. The dashed horizontal line (A) indicates the target FDR level of 0.05.

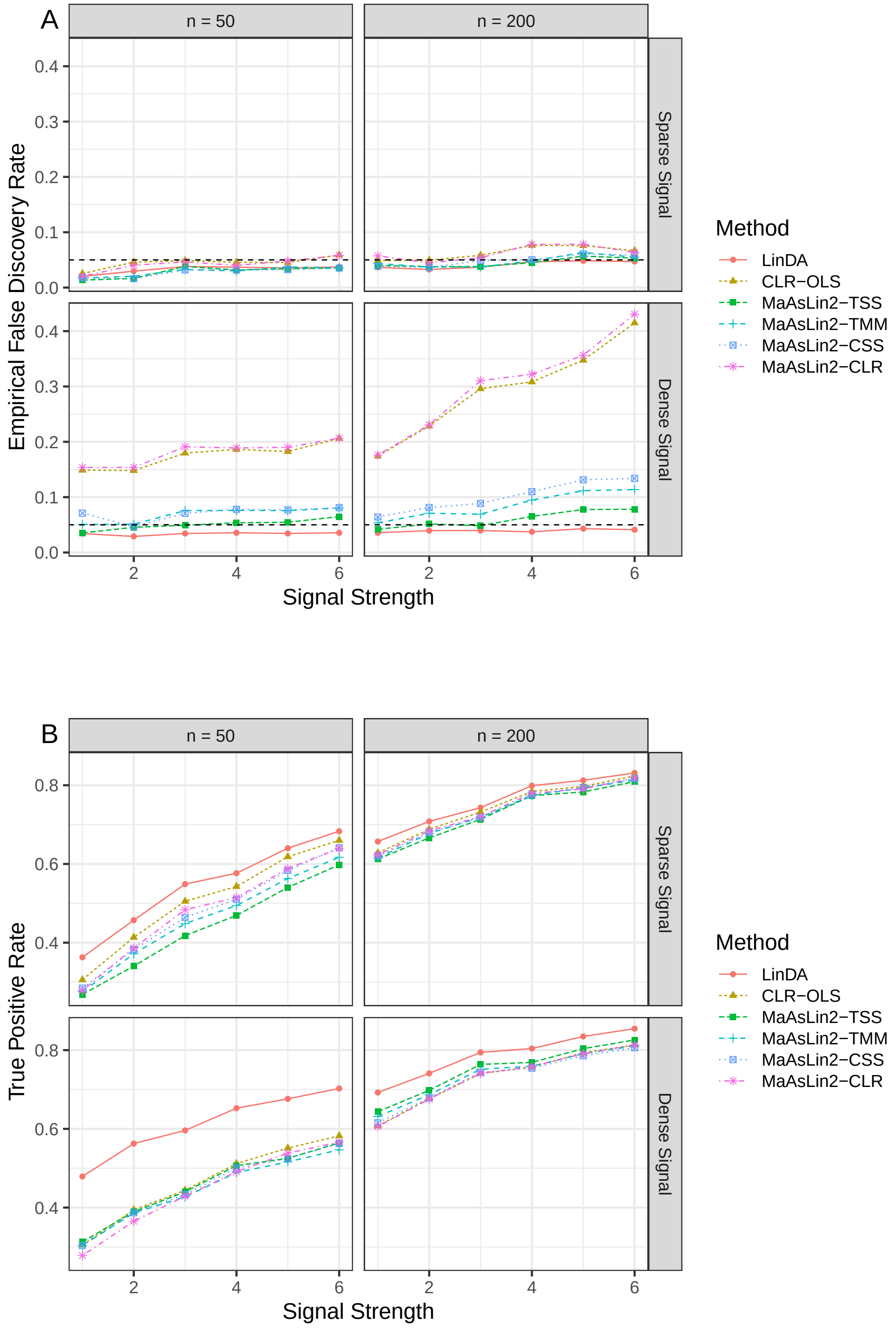

Fig. S3: Performance comparison between LinDA and MaAsLin2 (S0C0: log normal abundance distribution, a binary covariate). Empirical false discovery rate (A) and true positive rates (B) were averaged over 100 simulation runs. The dashed horizontal line (A) indicates the target FDR level of 0.05.

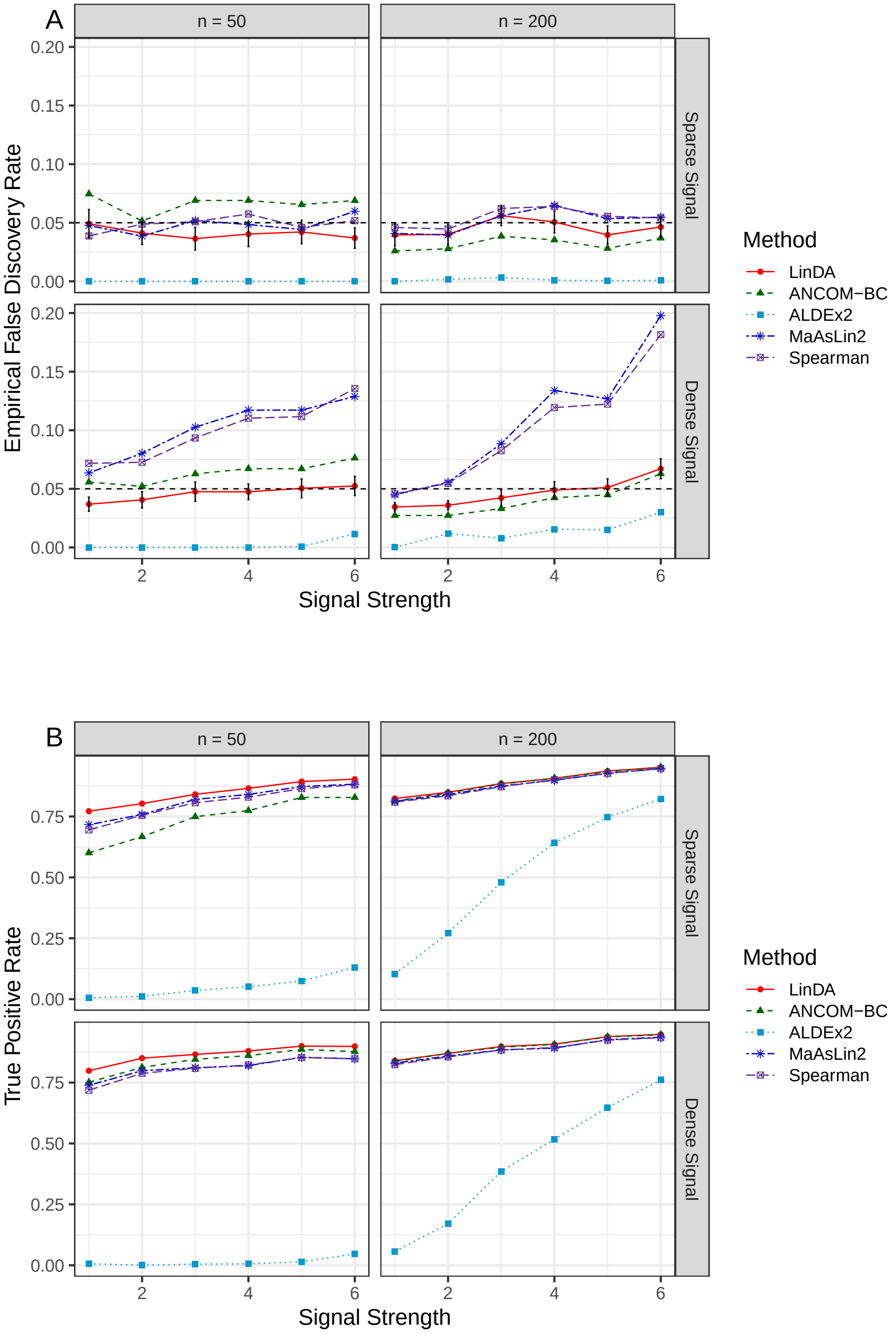

Fig. S4: Performance comparison (S0C1: log normal abundance distribution, a continuous covariate). Empirical false discovery rate (A) and true positive rates (B) were averaged over 100 simulation runs. Error bars (A) represent the 95% CIs of the method LinDA and the dashed horizontal line indicates the target FDR level of 0.05.

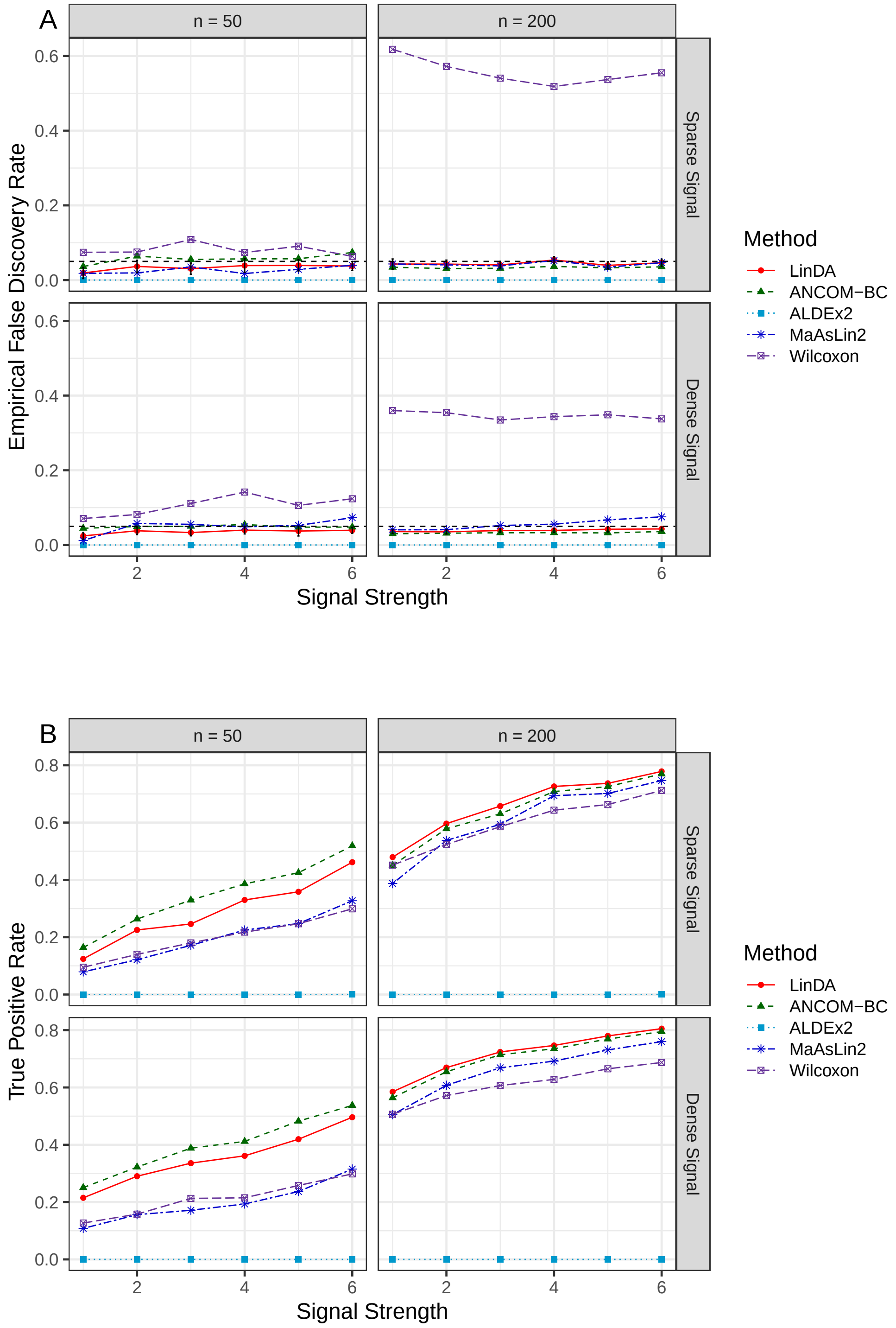

Fig. S5: Performance comparison (S0C2: log normal abundance distribution, a binary variable of interest and two confounders). Empirical false discovery rate (A) and true positive rates (B) were averaged over 100 simulation runs. Error bars (A) represent the 95% CIs of the method LinDA and the dashed horizontal line indicates the target FDR level of 0.05.

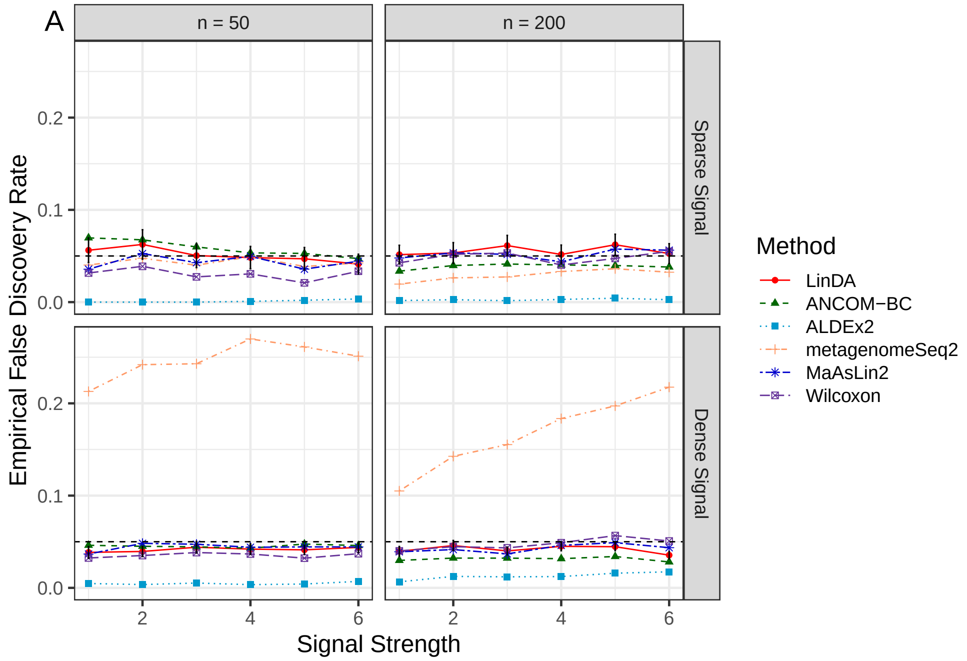

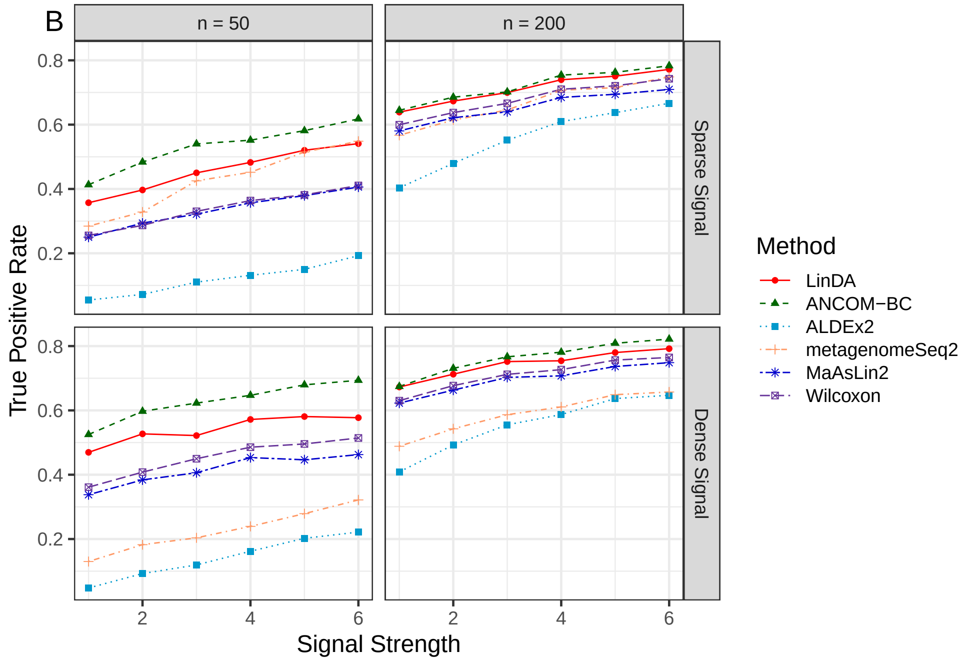

Fig. S6: Performance comparison (S1C0: zero inflated absolute abundances, a binary covariate). Empirical false discovery rate (A) and true positive rates (B) were averaged over 100 simulation runs. Error bars (A) represent the 95% CIs of the method LinDA and the dashed horizontal line indicates the target FDR level of 0.05.

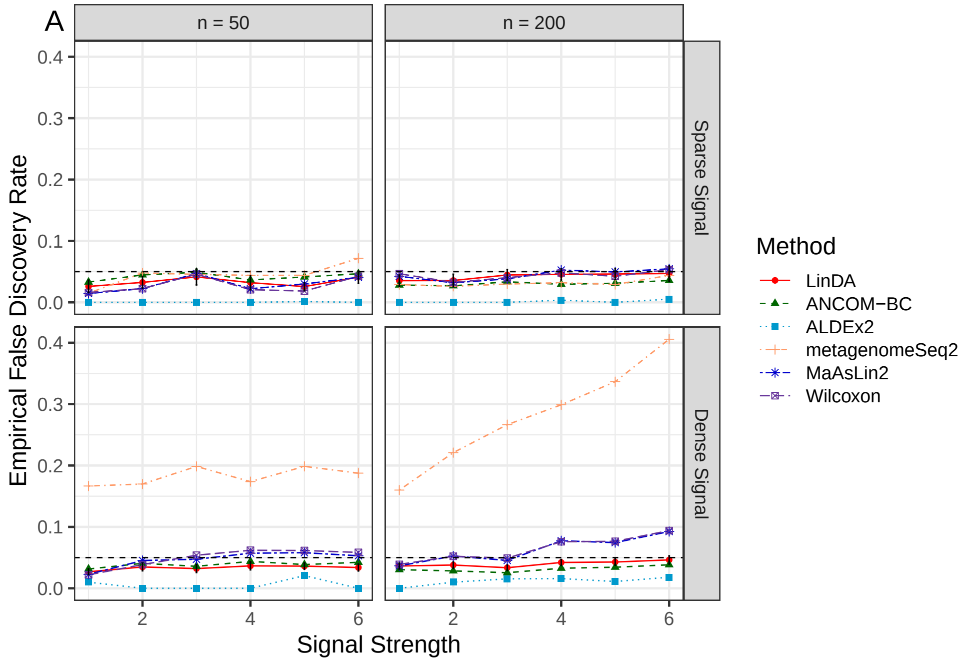

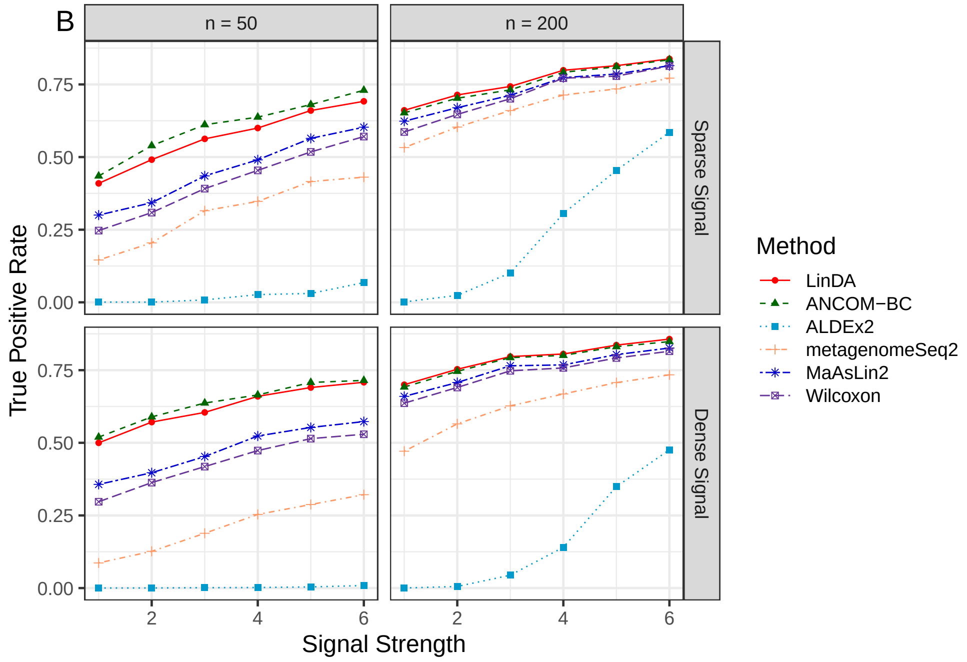

Fig. S7: Performance comparison (S2C0: correlated absolute abundances, a binary covariate). Empirical false discovery rate (A) and true positive rates (B) were averaged over 100 simulation runs. Error bars (A) represent the 95% CIs of the method LinDA and the dashed horizontal line indicates the target FDR level of 0.05.

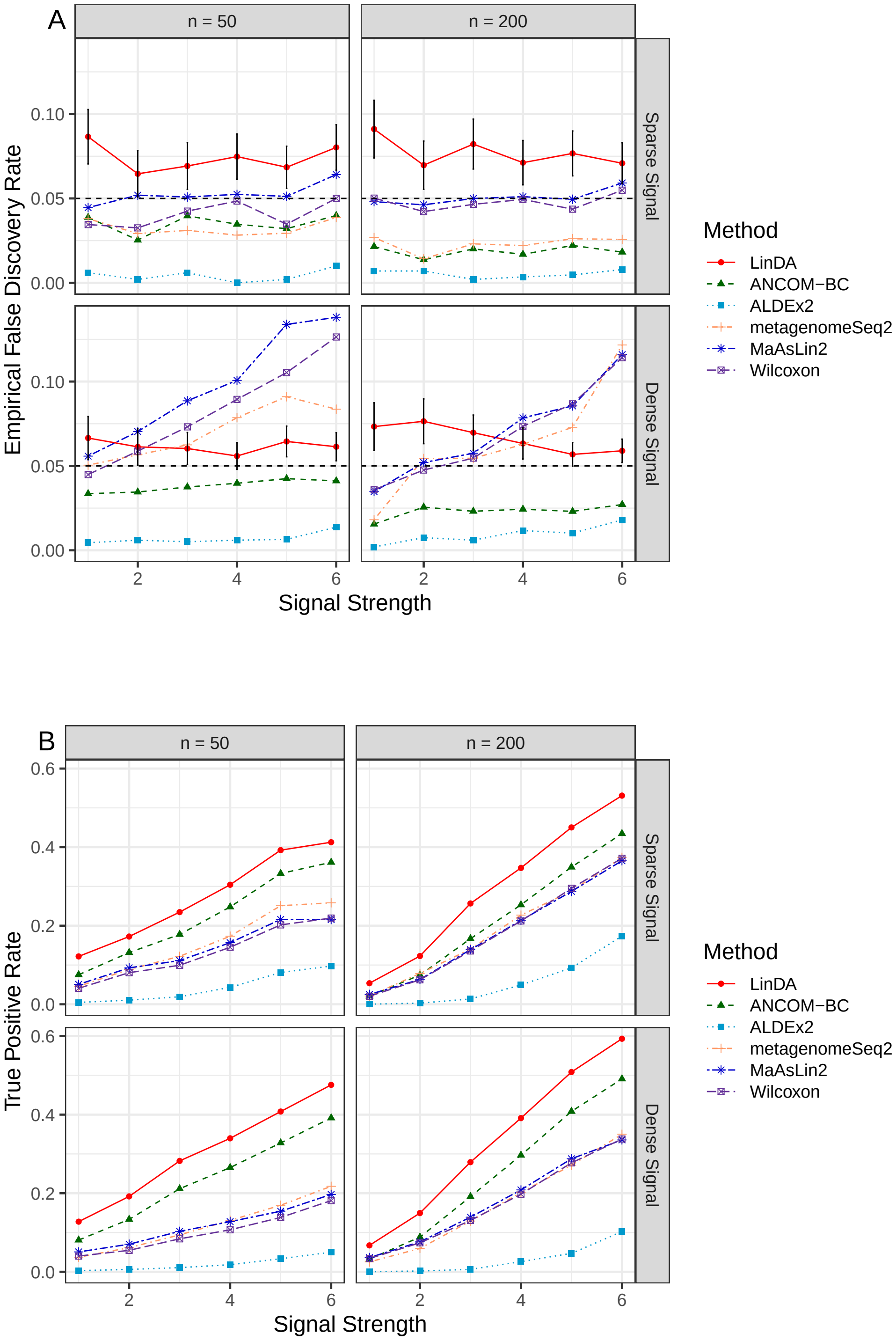

Fig. S8: Performance comparison (S4C0: smaller m, a binary covariate). Empirical false discovery rate (A) and true positive rates (B) were averaged over 1000 simulation runs. Error bars (A) represent the 95% CIs of the method LinDA and the dashed horizontal line indicates the target FDR level of 0.05.

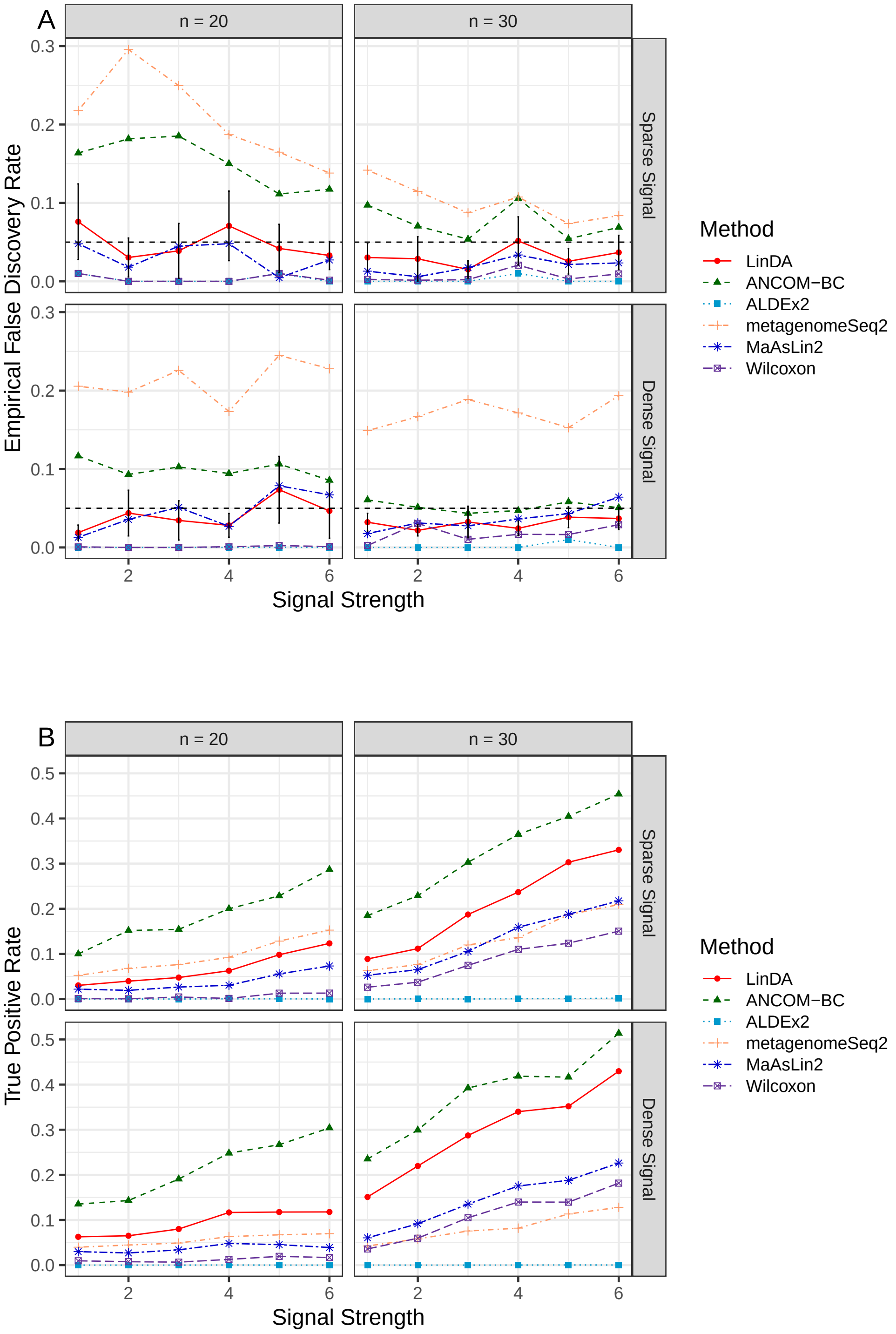

Fig. S9: Performance comparison (S5C0: smaller n, a binary covariate). Empirical false discovery rate (A) and true positive rates (B) were averaged over 100 simulation runs. Error bars (A) represent the 95% CIs of the method LinDA and the dashed horizontal line indicates the target FDR level of 0.05.

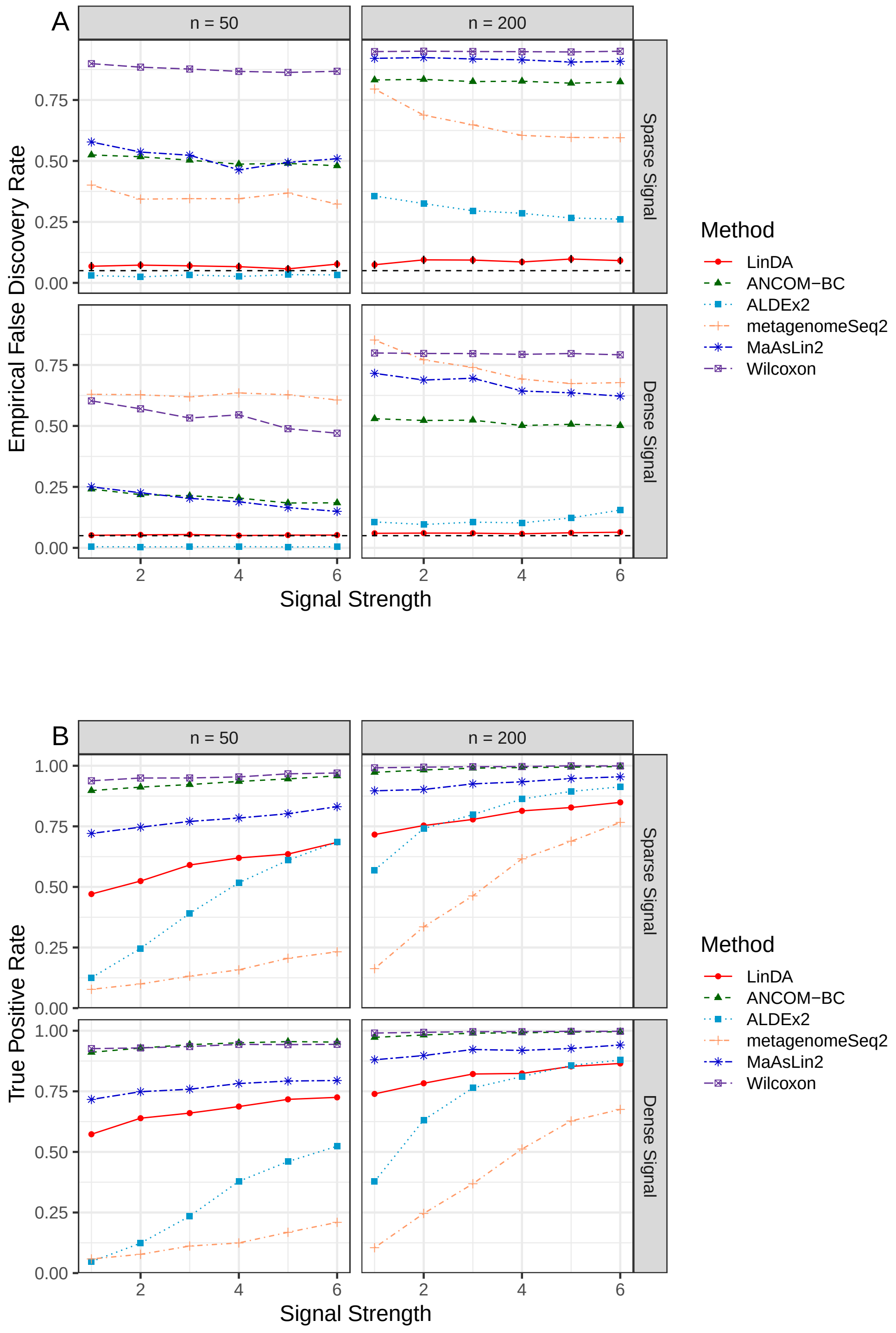

Fig. S10: Performance comparison (S6C0: 10-fold difference in library size, a binary covariate). Empirical false discovery rate (A) and true positive rates (B) were averaged over 100 simulation runs. Error bars (A) represent the 95% CIs of the method LinDA and the dashed horizontal line indicates the target FDR level of 0.05.

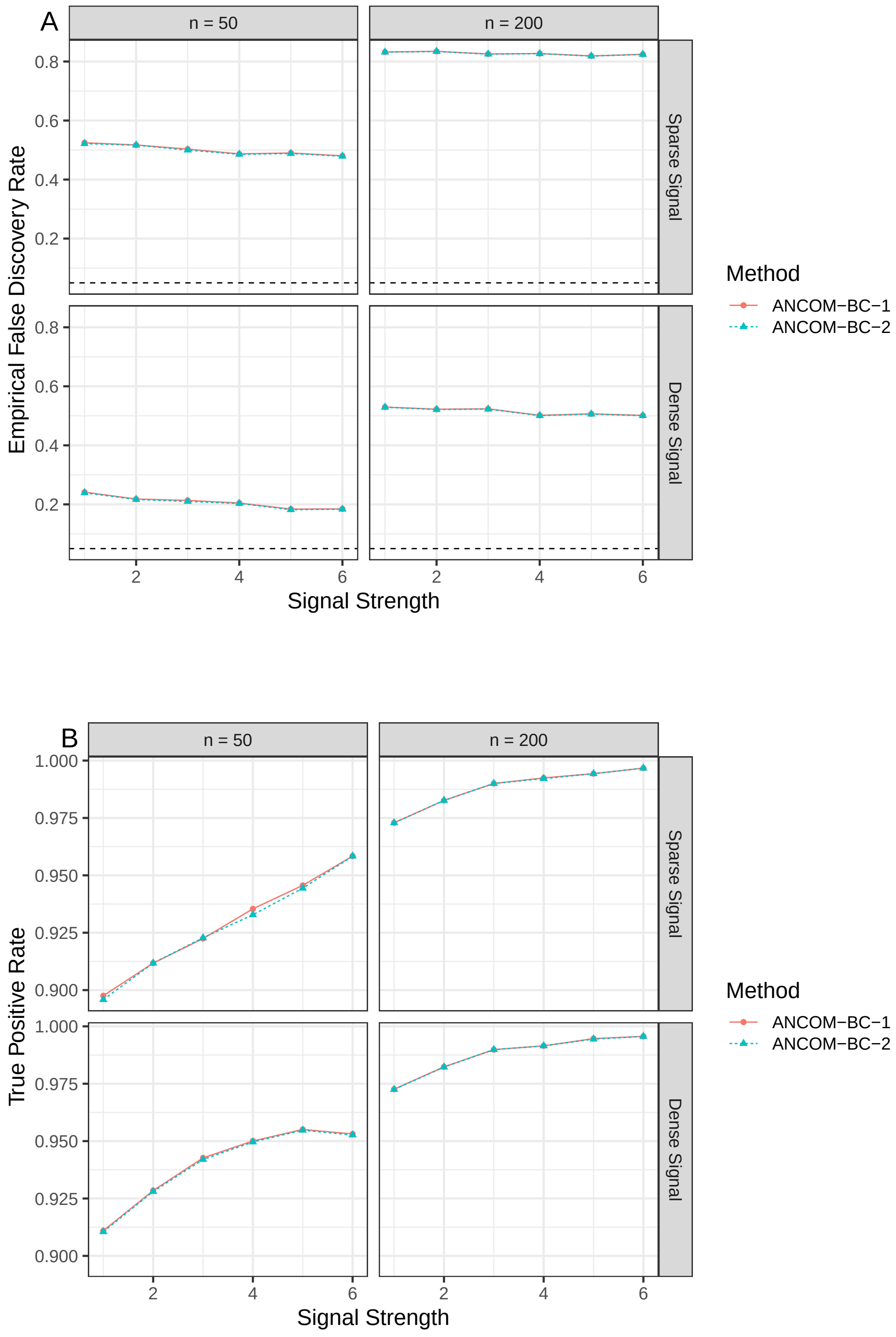

Fig. S11: Performance of ANCOM-BC disabling (ANCOM-BC-1) and enabling (ANCOM-BC-2) zero treatment (S6C0: 10-fold difference in library size, a binary covariate). Empirical false discovery rate (A) and true positive rates (B) were averaged over 100 simulation runs. The dashed horizontal line (A) indicates the target FDR level of 0.05.

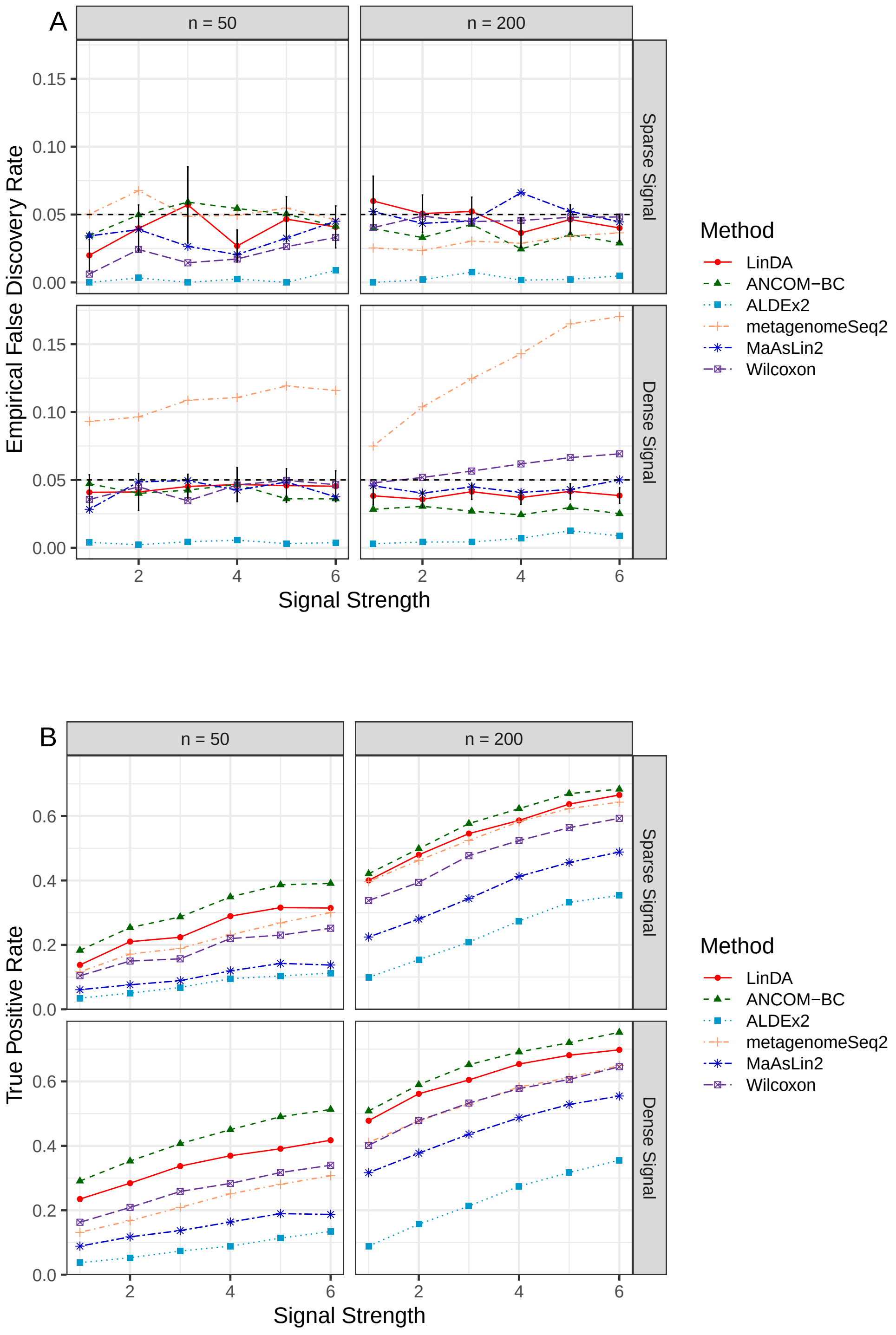

Fig. S12: Performance comparison (S7C0: negative binomial abundance distribution, a binary covariate). Empirical false discovery rate (A) and true positive rates (B) were averaged over 100 simulation runs. Error bars (A) represent the 95% CIs of the method LinDA and the dashed horizontal line indicates the target FDR level of 0.05.

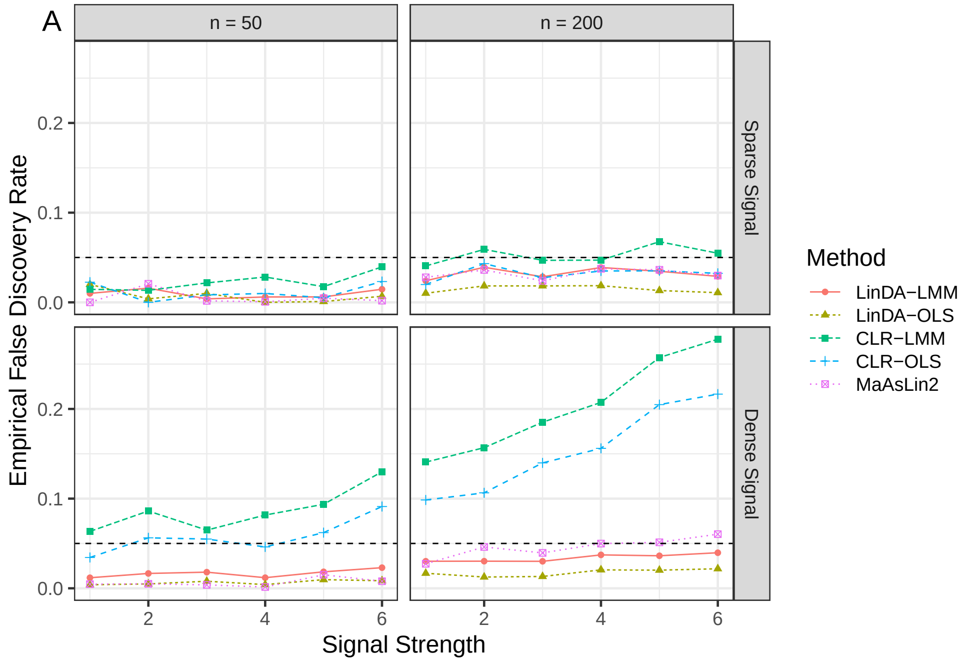

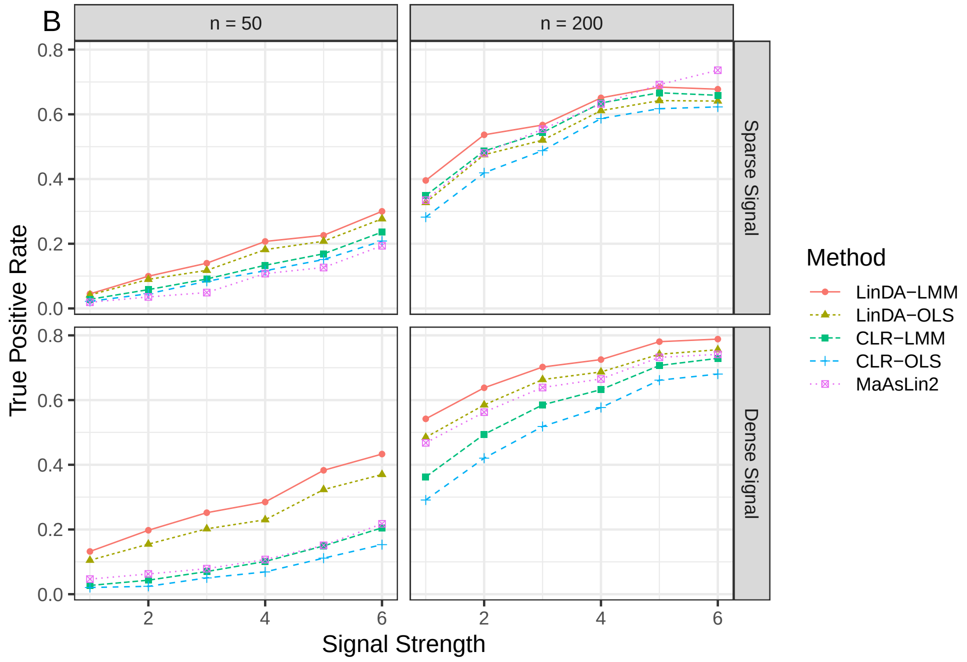

Fig. S13: Performance comparison (S8.1C0: pre-treatment and post-treatment comparison, a binary covariate). Empirical false discovery rate (A) and true positive rates (B) were averaged over 100 simulation runs. The dashed horizontal line (A) indicates the target FDR level of 0.05.

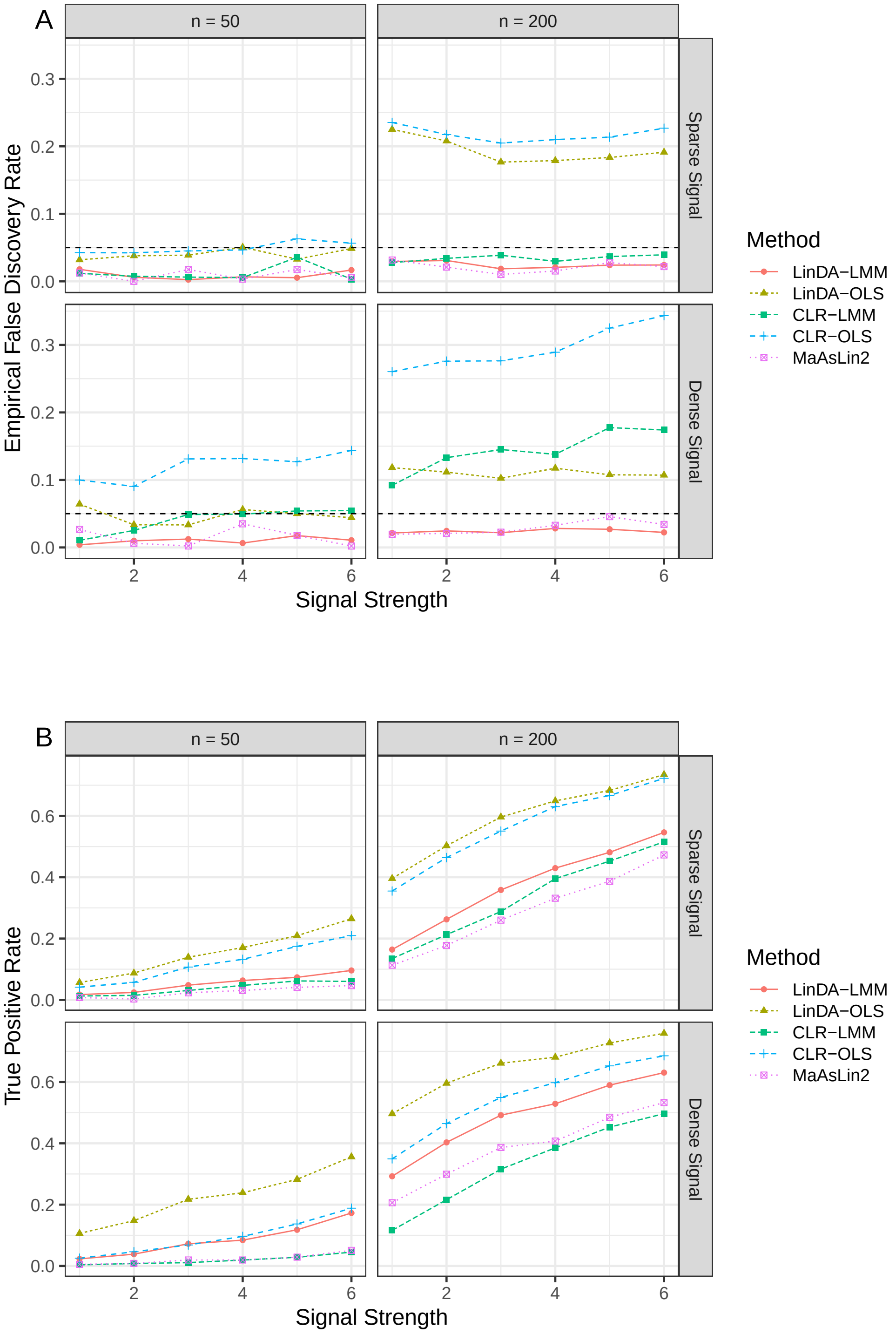

Fig. S14: Performance comparison (S8.2C0: replicate sampling, a binary covariate). Empirical false discovery rate (A) and true positive rates (B) were averaged over 100 simulation runs. The dashed horizontal line (A) indicates the target FDR level of 0.05.

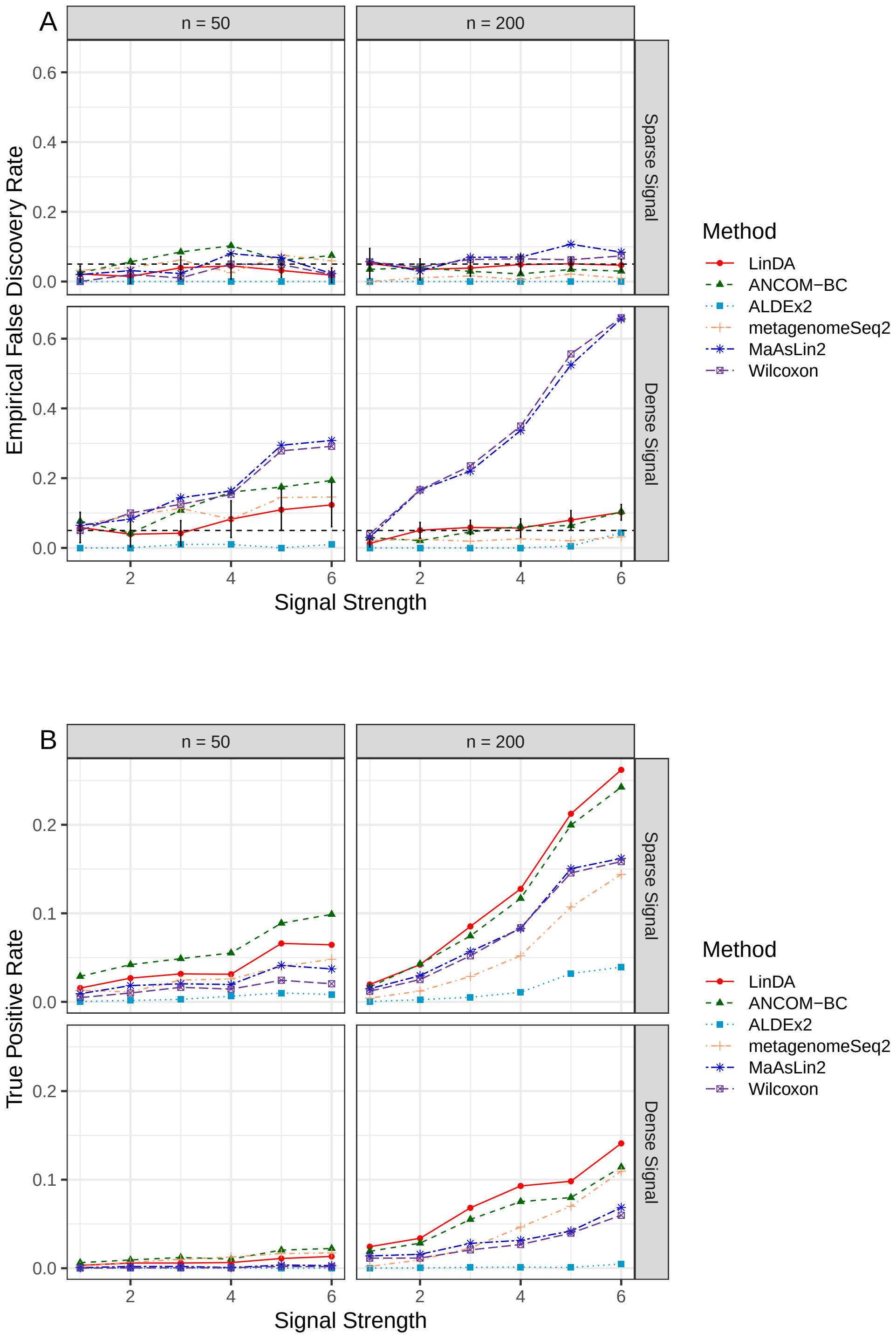

Fig. S15: Performance comparison (S0C0 with strong compositional effects). Empirical false discovery rate (A) and true positive rates (B) were averaged over 100 simulation runs. Error bars (A) represent the 95% CIs of the method LinDA and the dashed horizontal line indicates the target FDR level of 0.05.

A

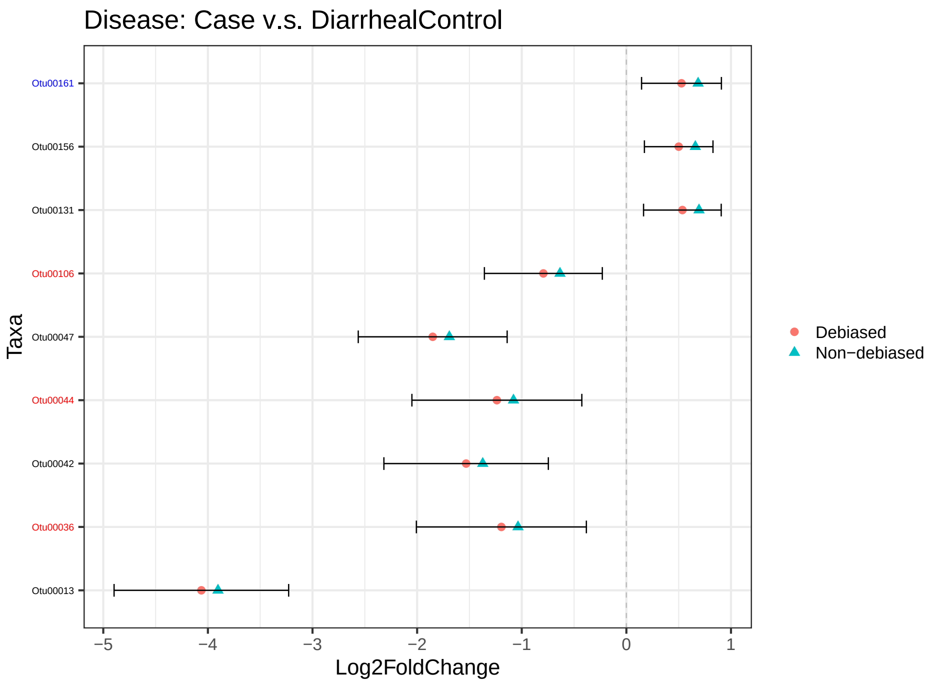

B

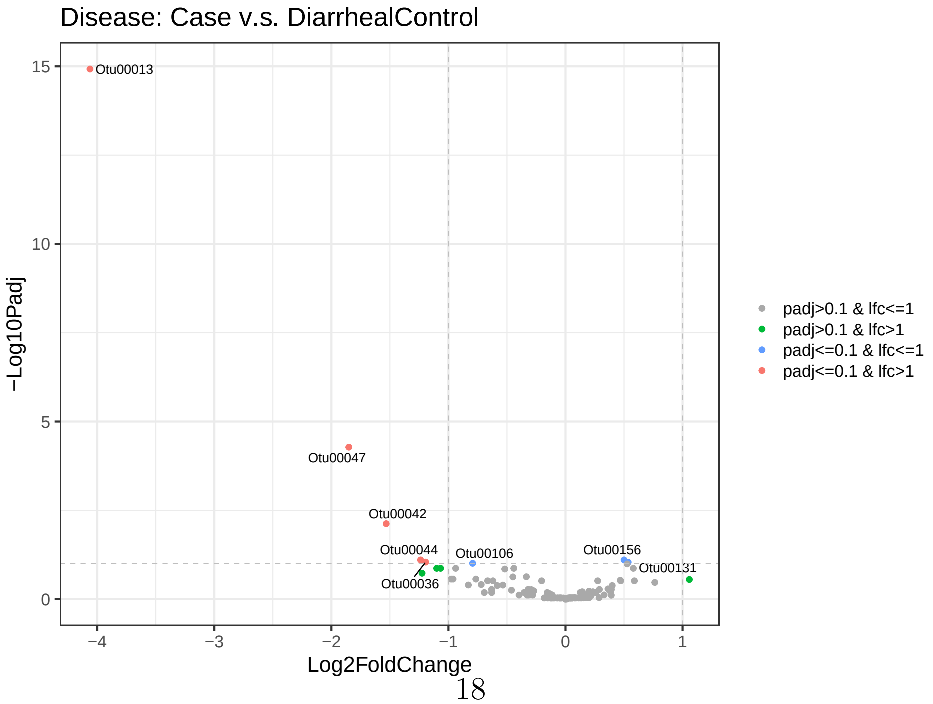

Fig. S16: Effect size plot (A) of differential taxa at FDR level of 0.1 and volcano plot (B) for the CDI dataset. The “Debiased” points represent the bias-corrected regression coefficients, and “Non-debiased” points represent the original (biased) regression coefficients. The error bars represent the 95% CIs of the “Debiased” points. The taxa in black are detected by LinDA, taxa in red are detected solely by LinDA, and the taxa in blue are missed by LinDA but detected by one or more of the other methods (A).

A

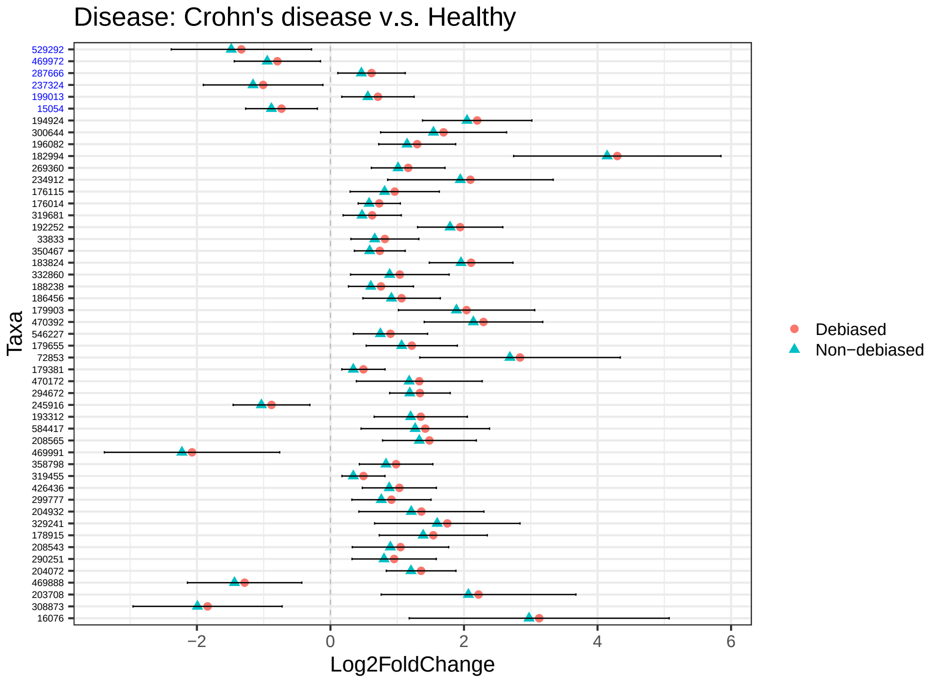

B

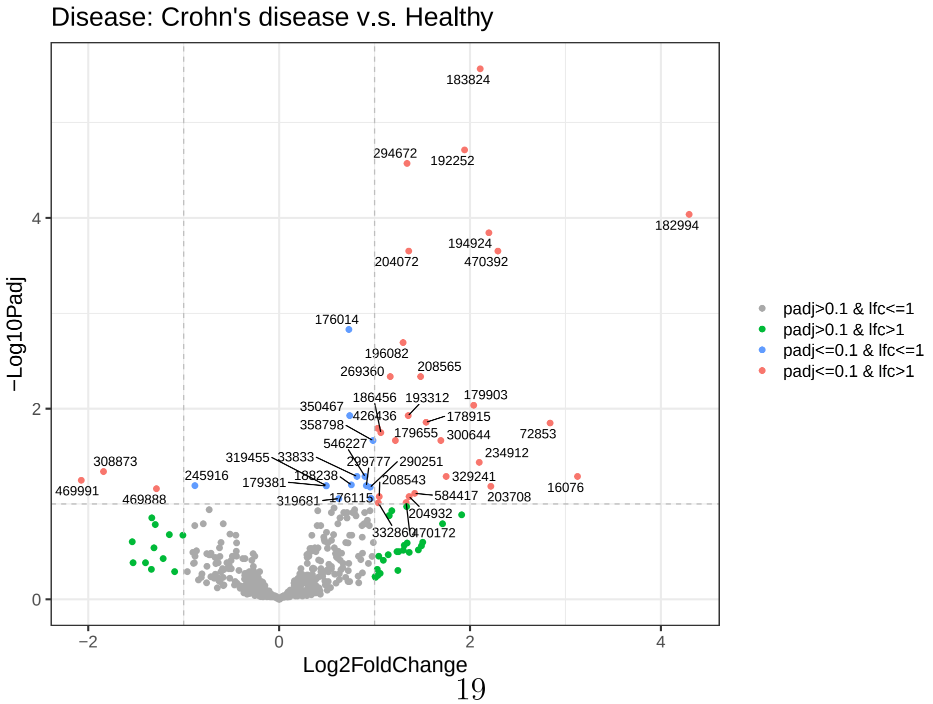

Fig. S17: Effect size plot (A) of differential taxa at FDR level of 0.1 and volcano plot (B) for the IBD dataset. The "Debiased" points represent the bias-corrected regression coefficients, and "Non-debiased" points represent the original (biased) regression coefficients. The error bars represent the 95% CIs of the "Debiased" points. The taxa in black are detected by LinDA, taxa in red are detected solely by LinDA, and taxa in blue are missed by LinDA but detected by two or more of the other methods (A).

A

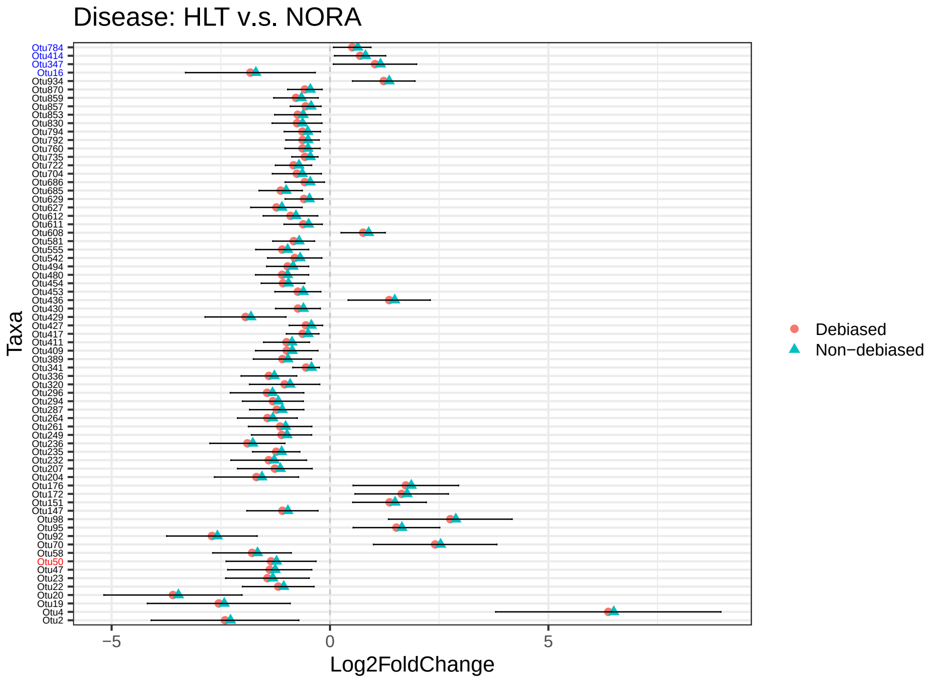

B

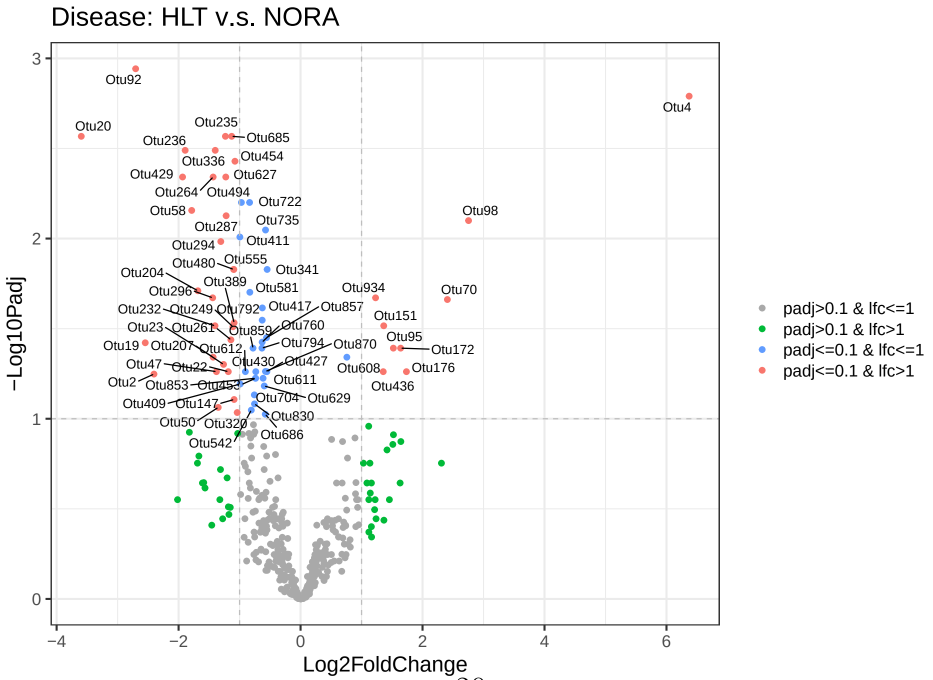

Fig. S18: Effect size plot (A) of differential taxa at FDR level of 0.1 and volcano plot (B) for the RA dataset. The “Debiased” points represent the bias-corrected regression coefficients, and “Non-debiased” points represent the original (biased) regression coefficients. The error bars represent the 95% CIs of the “Debiased” points. The taxa in black are detected by LinDA, taxa in red are detected solely by LinDA, and taxa in blue are missed by LinDA but detected by two or more of the other methods (A).

A

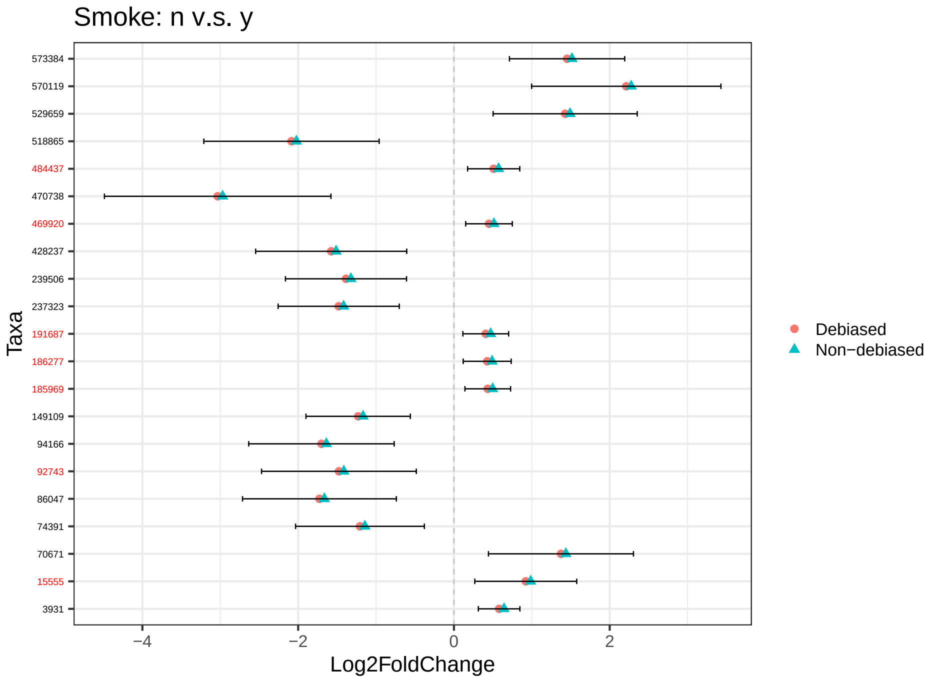

B

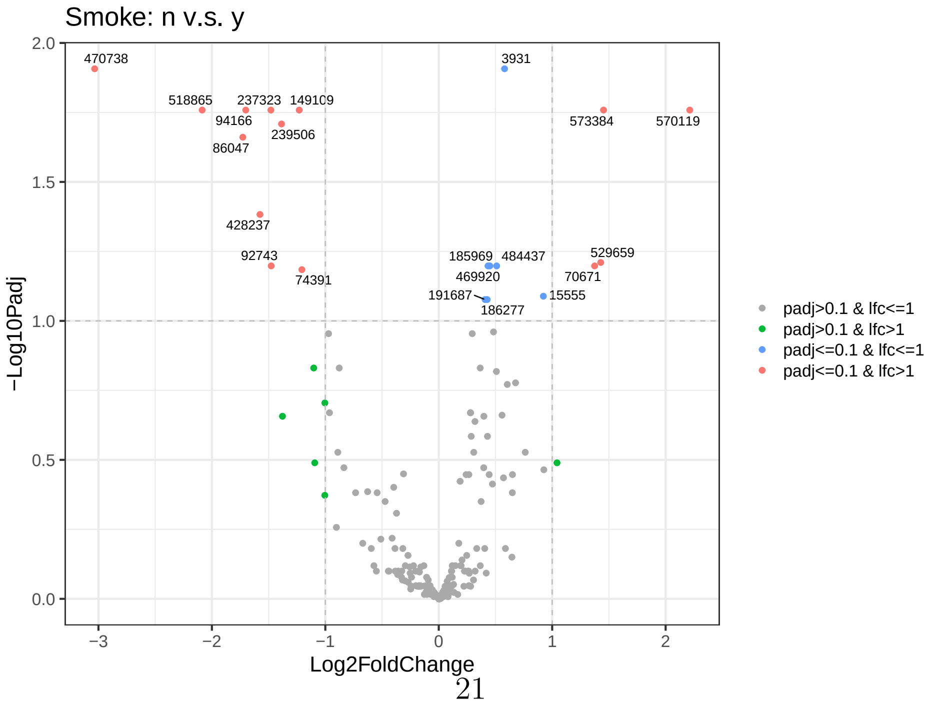

Fig. S19: Effect size plot (A) of differential taxa detected by LinDA at FDR level of 0.1 and volcano plot (B) for the SMOKE dataset. The "Debiased" points represent the bias-corrected regression coefficients, and "Non-debiased" points represent the original (biased) regression coefficients. The error bars represent the 95% CIs of the "Debiased" points. The taxa in black are detected by LinDA, taxa in red are detected by LinDA but missed by MaAsLin2, and no taxa are detected by MaAsLin2 but missed by LinDA (A).

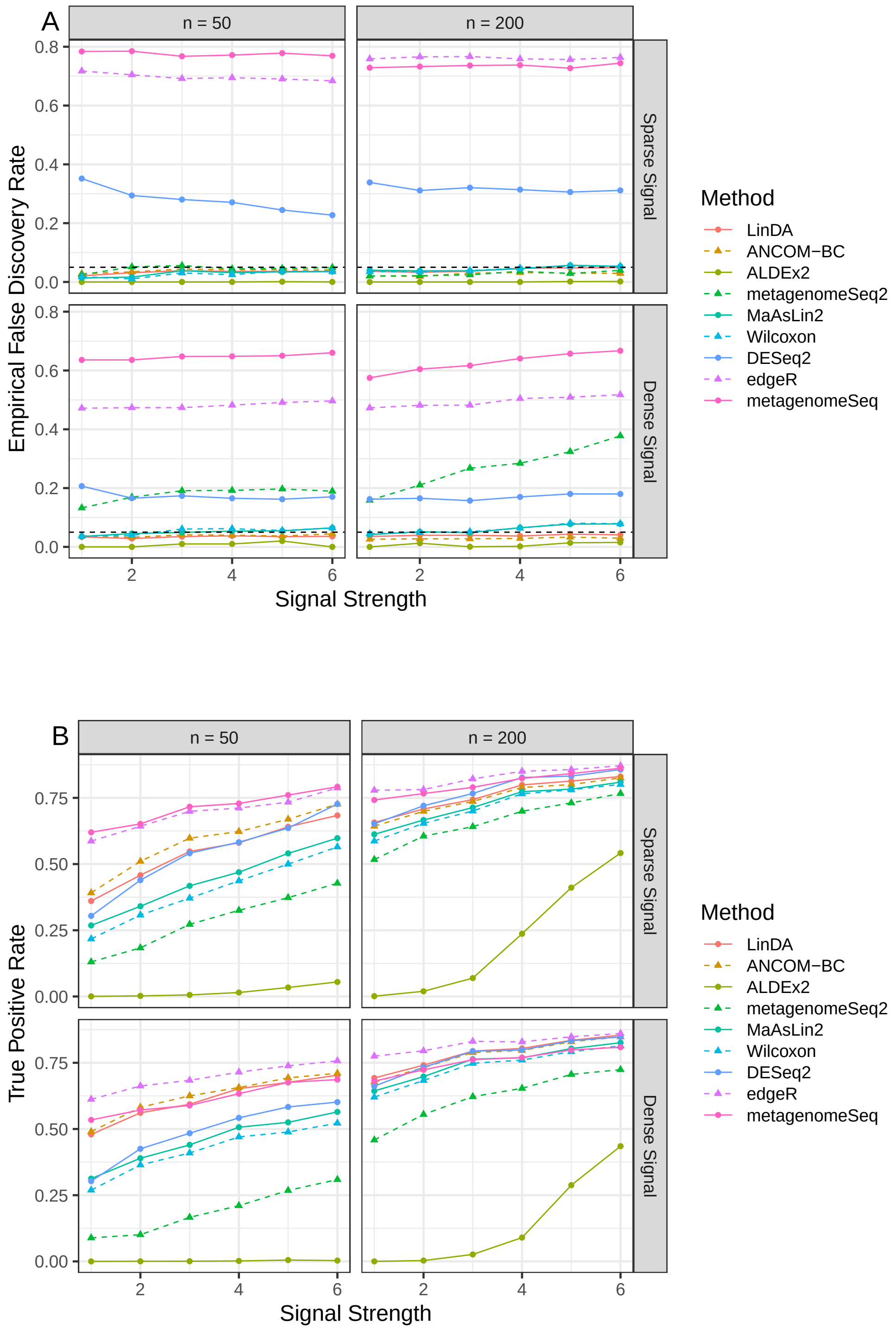

Fig. S20: Full performance comparison (S0C0: log normal abundance distribution, a binary covariate). Empirical false discovery rate (A) and true positive rates (B) were averaged over 100 simulation runs. The dashed horizontal line (A) indicates the target FDR level of 0.05.

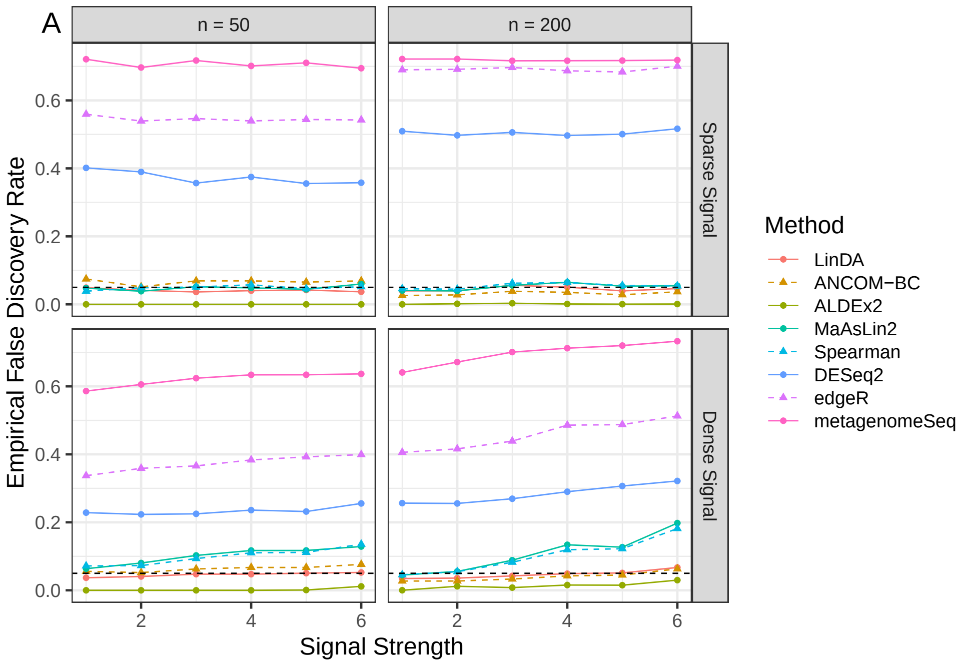

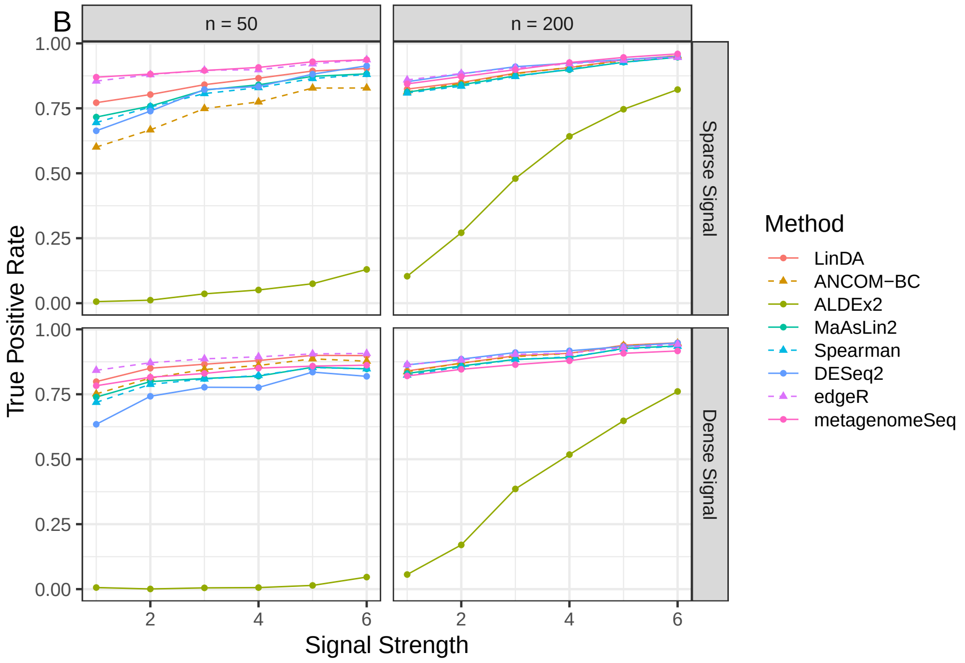

Fig. S21: Full performance comparison (S0C1: log normal abundance distribution, a continuous covariate). Empirical false discovery rate (A) and true positive rates (B) were averaged over 100 simulation runs. The dashed horizontal line (A) indicates the target FDR level of 0.05.

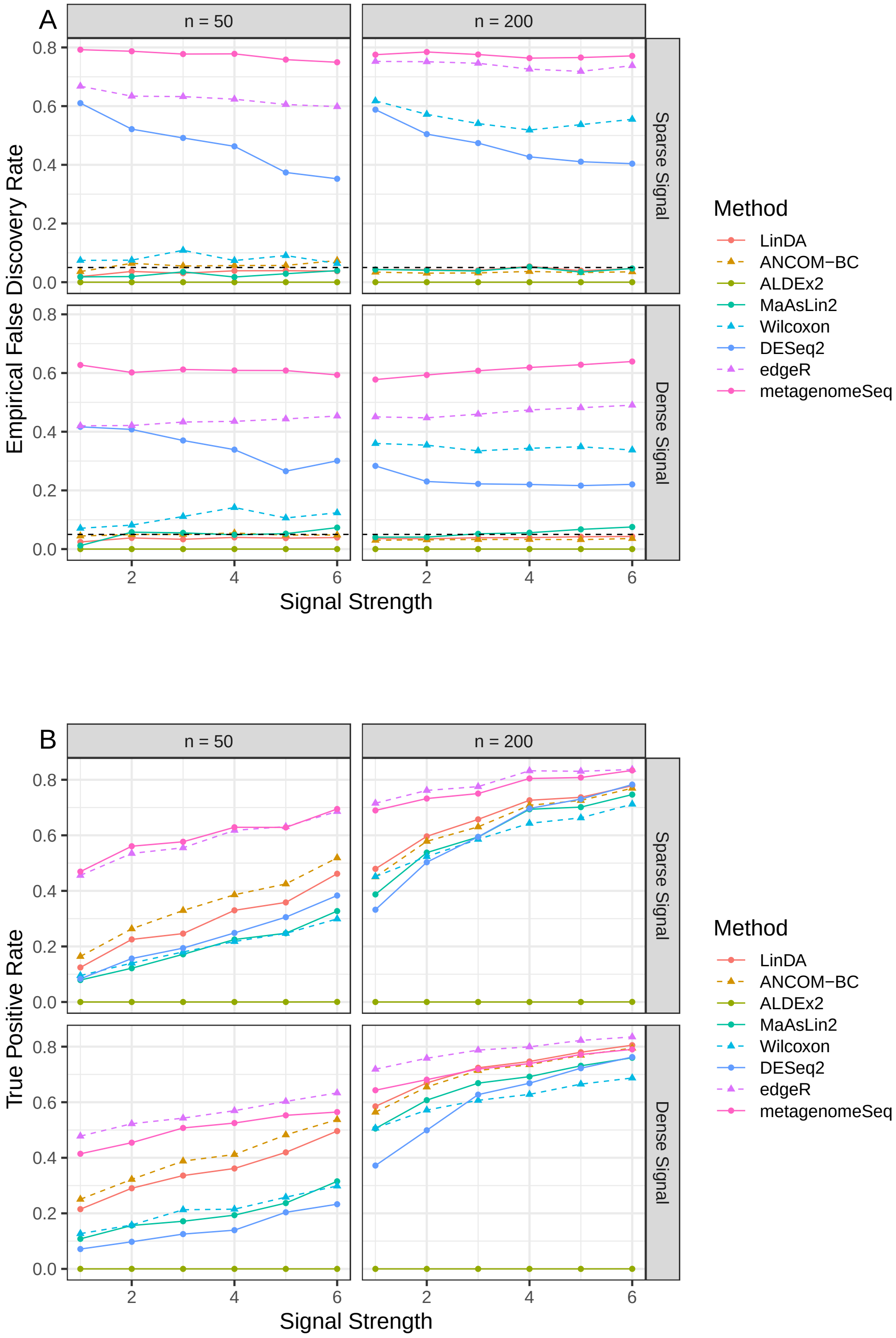

Fig. S22: Full performance comparison (S0C2: log normal abundance distribution, a binary variable of interest and two confounders). Empirical false discovery rate (A) and true positive rates (B) were averaged over 100 simulation runs. The dashed horizontal line (A) indicates the target FDR level of 0.05.

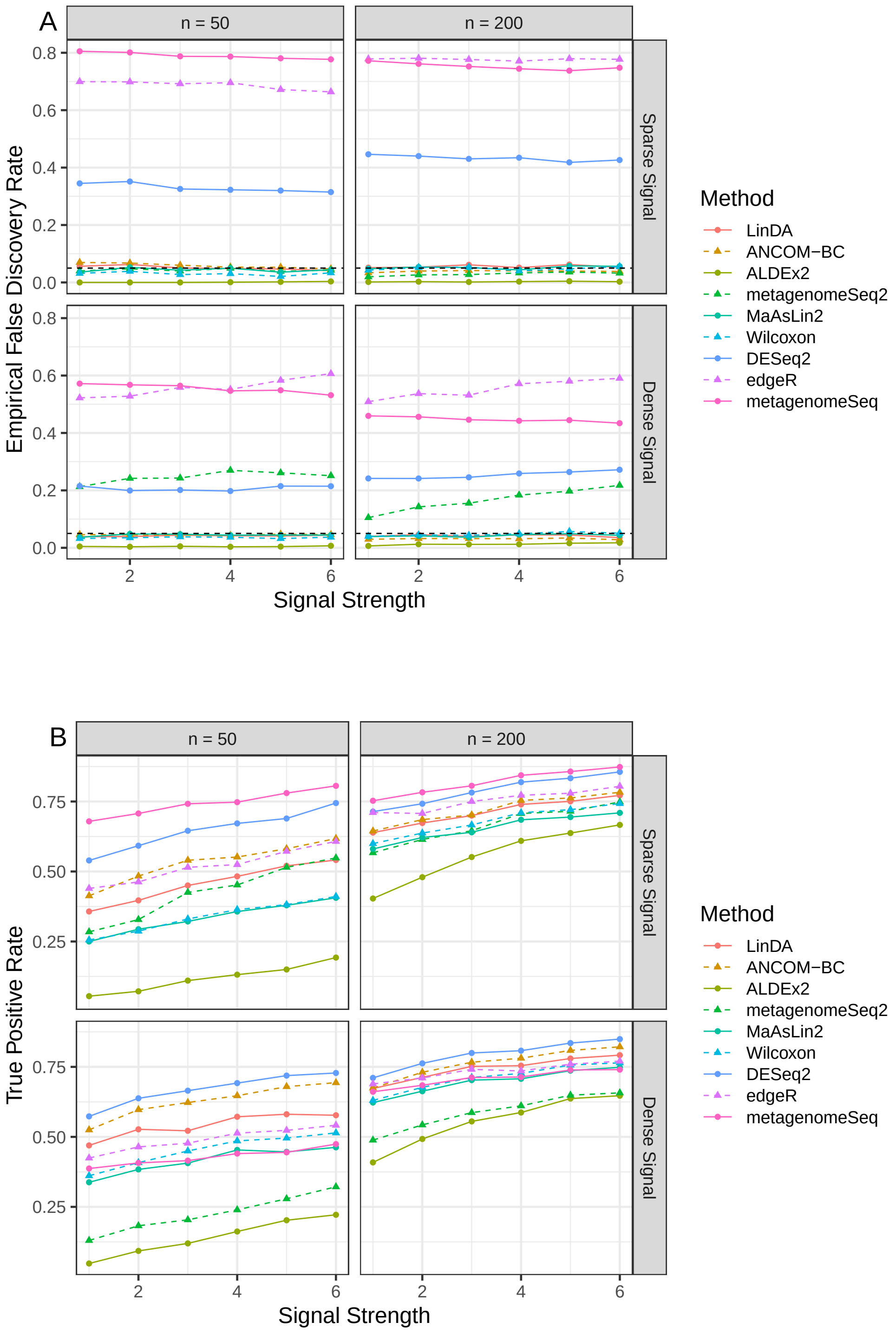

Fig. S23: Full performance comparison (S1C0: zero inflated absolute abundances, a binary covariate). Empirical false discovery rate (A) and true positive rates (B) were averaged over 100 simulation runs. The dashed horizontal line (A) indicates the target FDR level of 0.05.

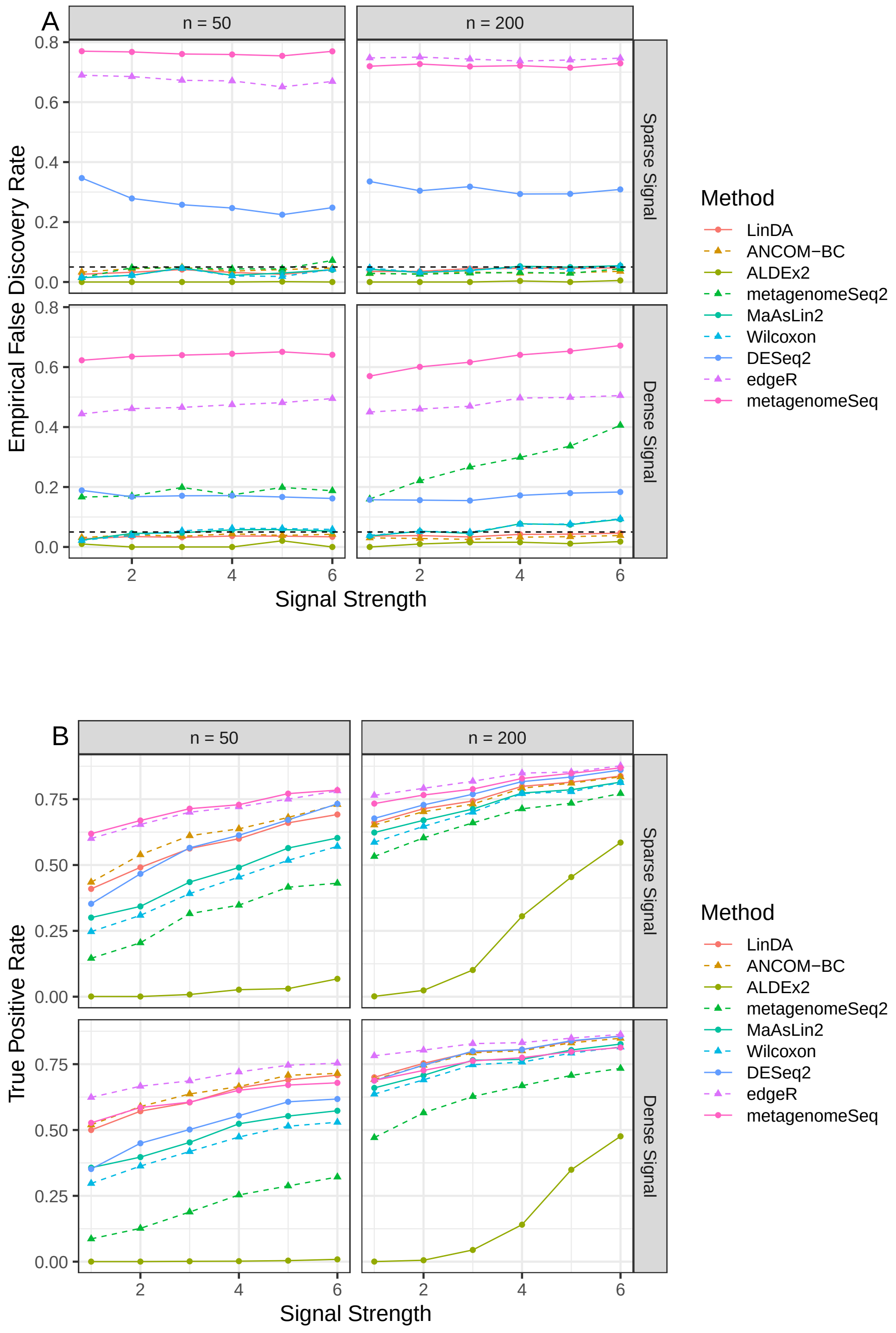

Fig. S24: Full performance comparison (S2C0: correlated absolute abundances, a binary covariate). Empirical false discovery rate (A) and true positive rates (B) were averaged over 100 simulation runs. The dashed horizontal line (A) indicates the target FDR level of 0.05.

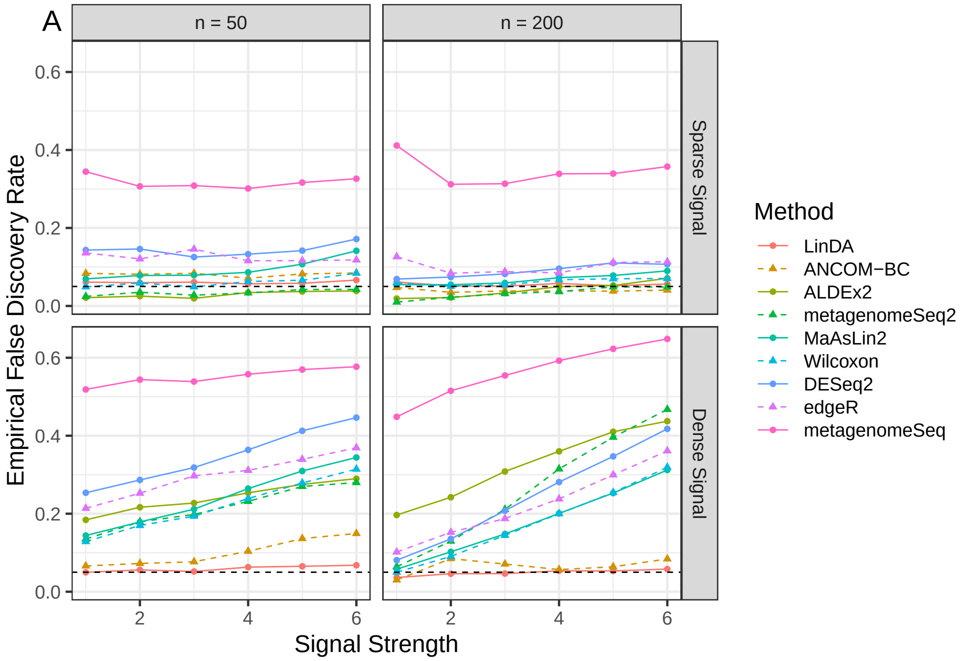

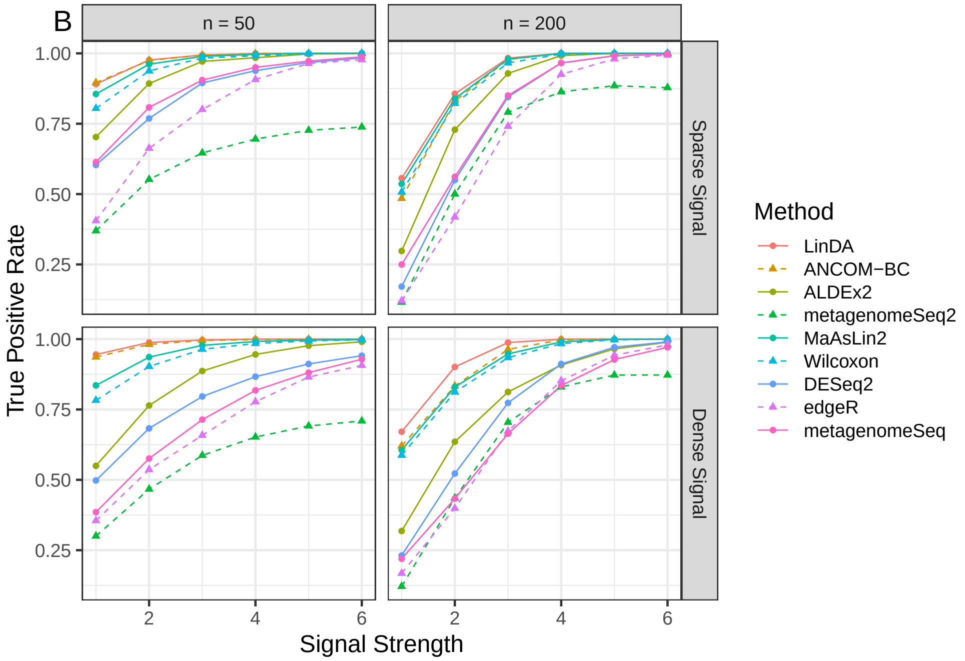

Fig. S25: Full performance comparison (S3C0: gamma abundance distribution, a binary covariate). Empirical false discovery rate (A) and true positive rates (B) were averaged over 100 simulation runs. The dashed horizontal line (A) indicates the target FDR level of 0.05.

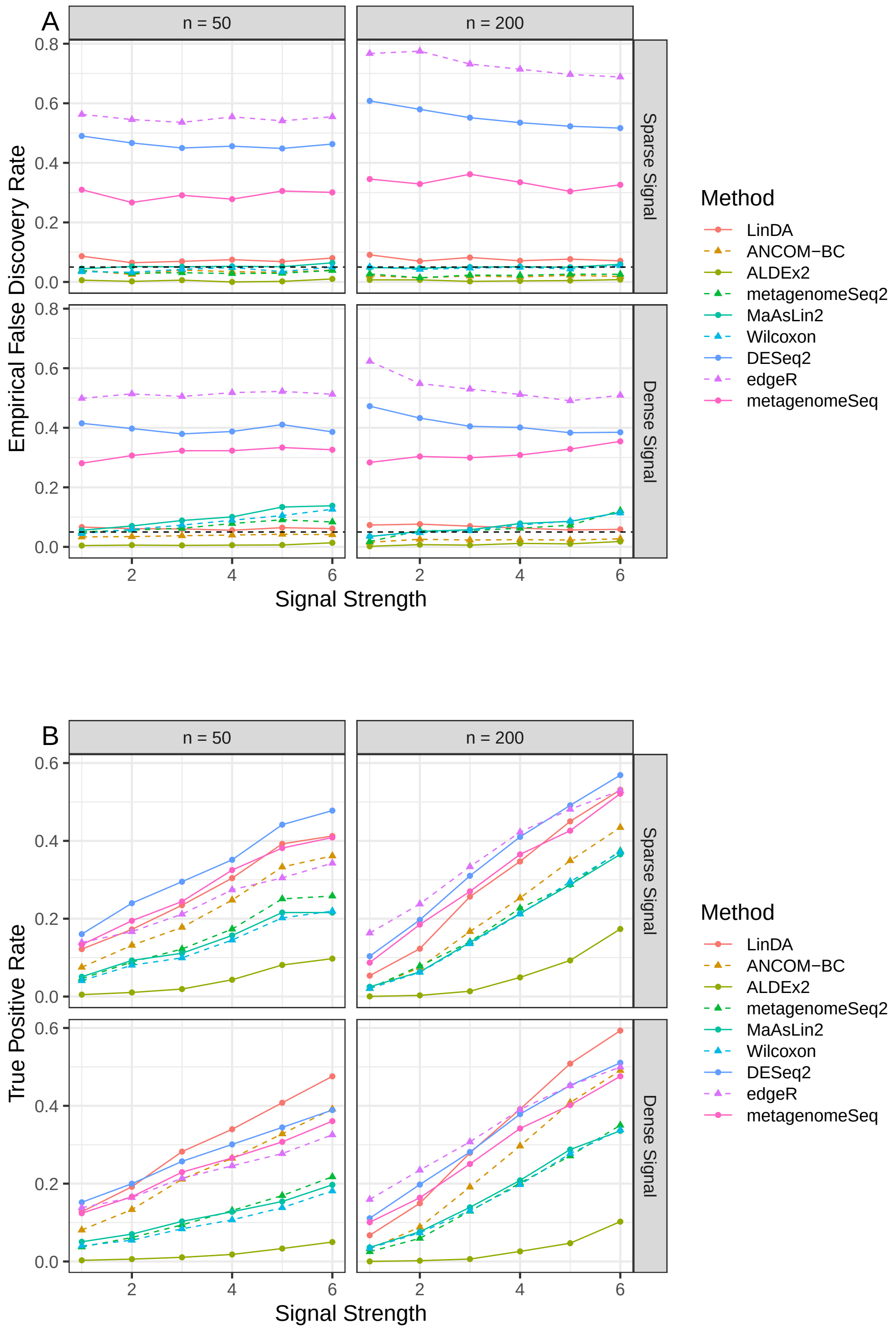

Fig. S26: Full performance comparison (S4C0: smaller m, a binary covariate). Empirical false discovery rate (A) and true positive rates (B) were averaged over 1000 simulation runs. The dashed horizontal line (A) indicates the target FDR level of 0.05.

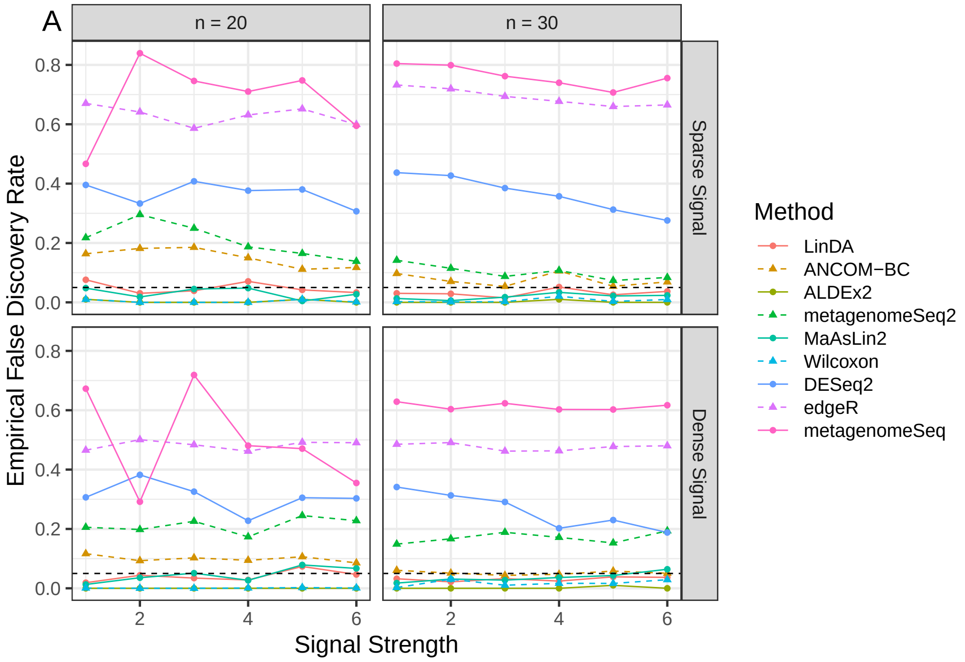

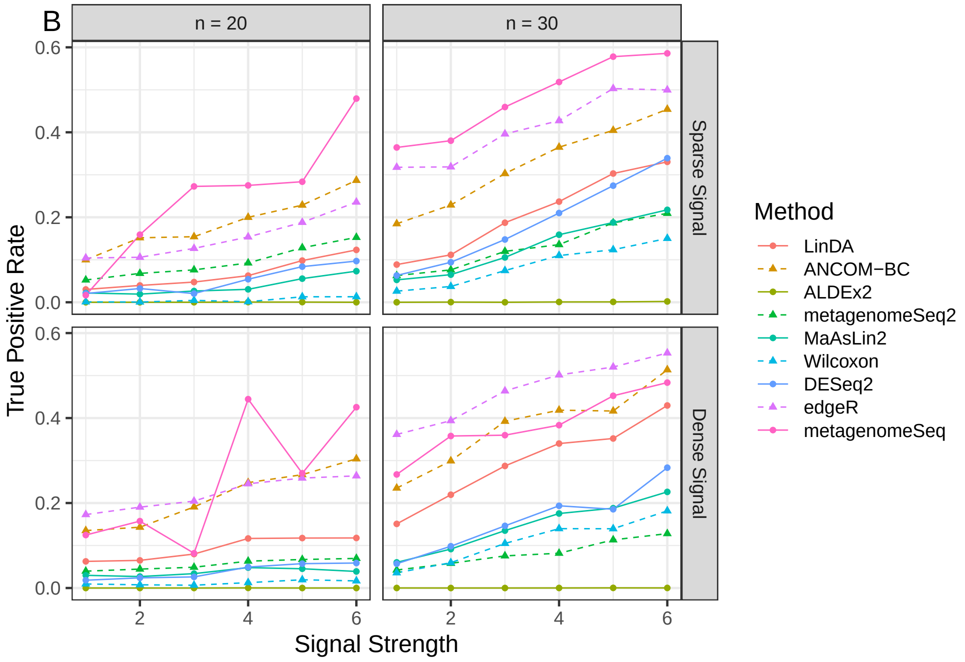

Fig. S27: Full performance comparison (S5C0: smaller n, a binary covariate). Empirical false discovery rate (A) and true positive rates (B) were averaged over 100 simulation runs. The dashed horizontal line (A) indicates the target FDR level of 0.05.

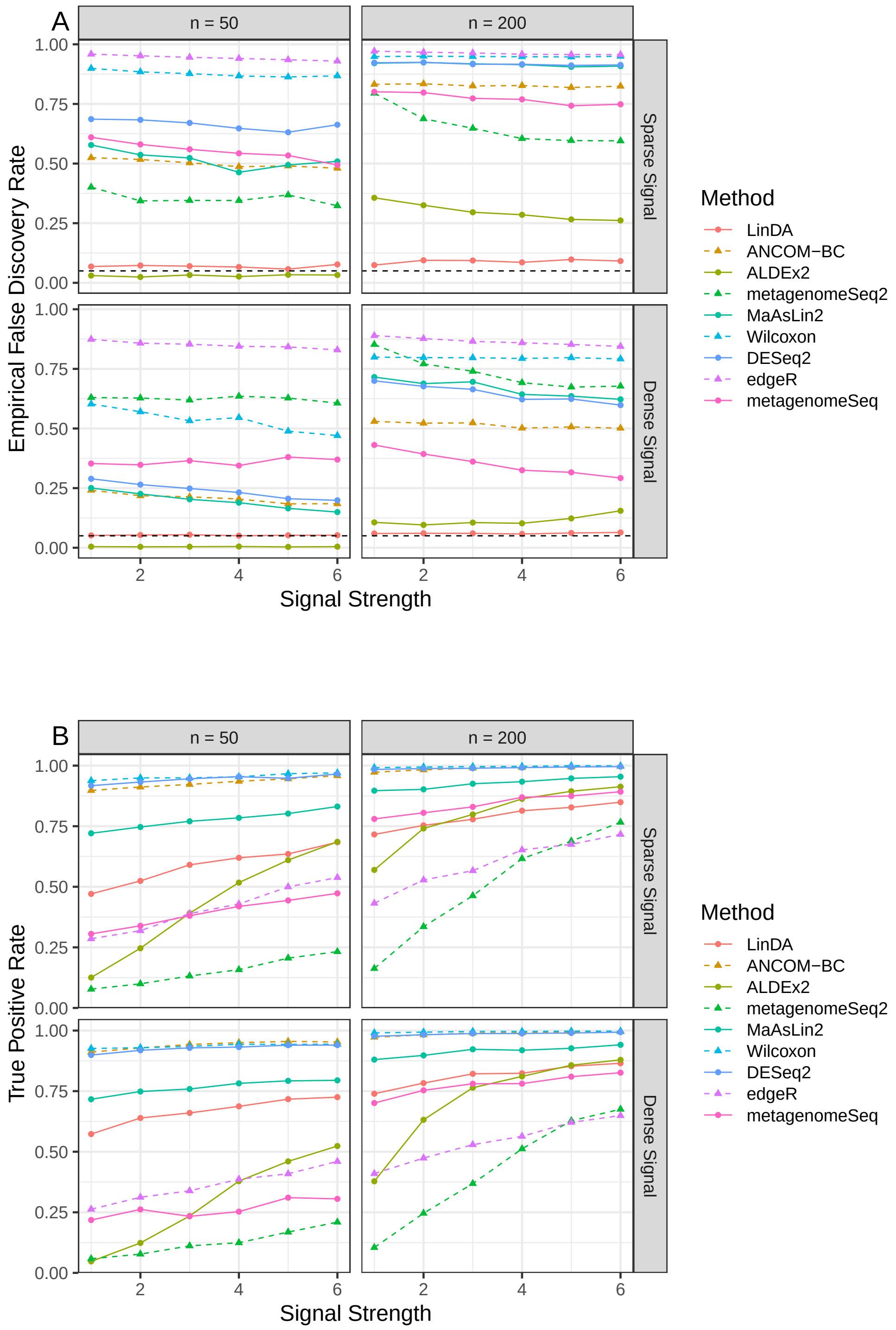

Fig. S28: Full performance comparison (S6C0: 10-fold difference in library size, a binary covariate). Empirical false discovery rate (A) and true positive rates (B) were averaged over 100 simulation runs. The dashed horizontal line (A) indicates the target FDR level of 0.05.

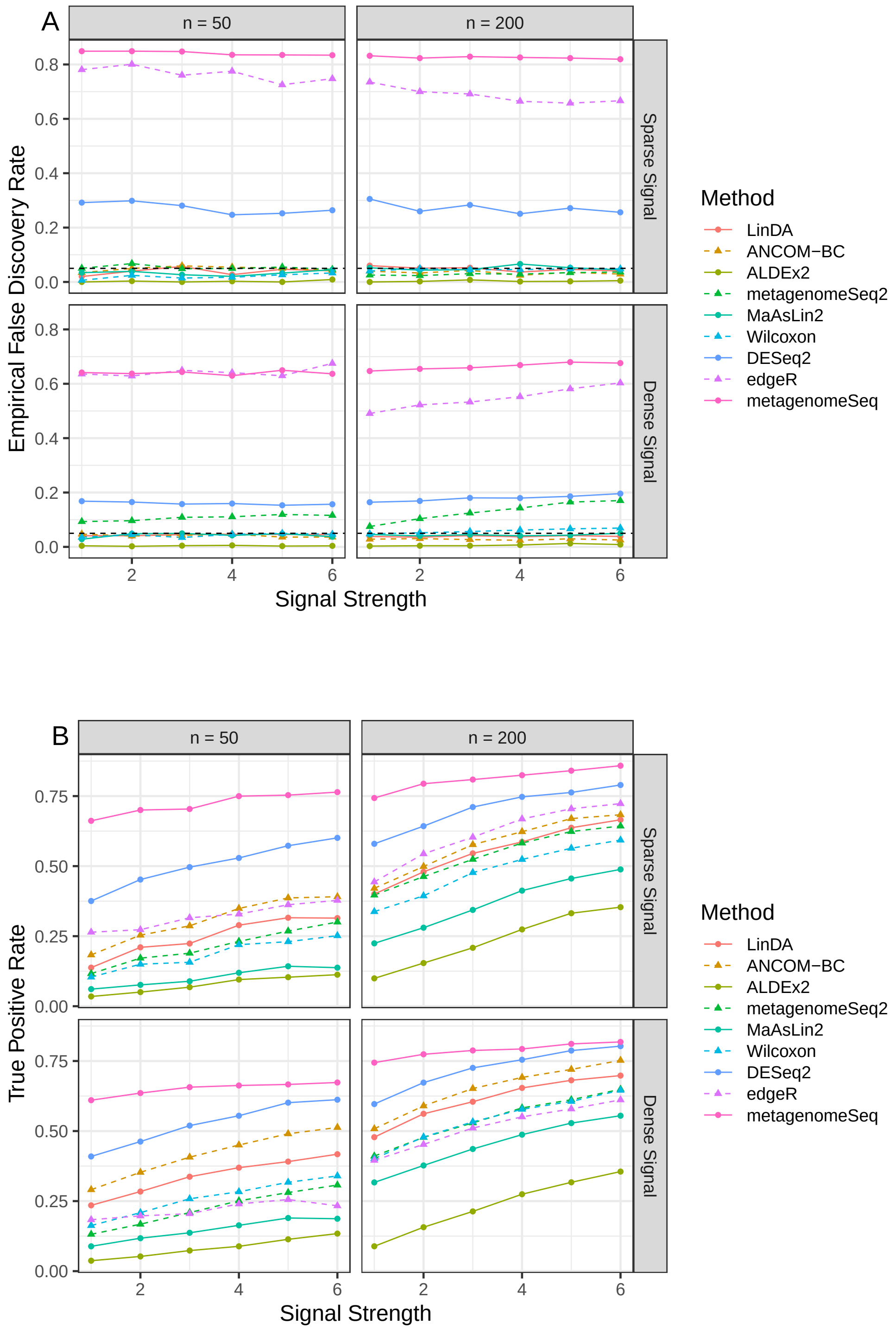

Fig. S29: Full performance comparison (S7C0: negative binomial abundance distribution, a binary covariate). Empirical false discovery rate (A) and true positive rates (B) were averaged over 100 simulation runs. The dashed horizontal line (A) indicates the target FDR level of 0.05.

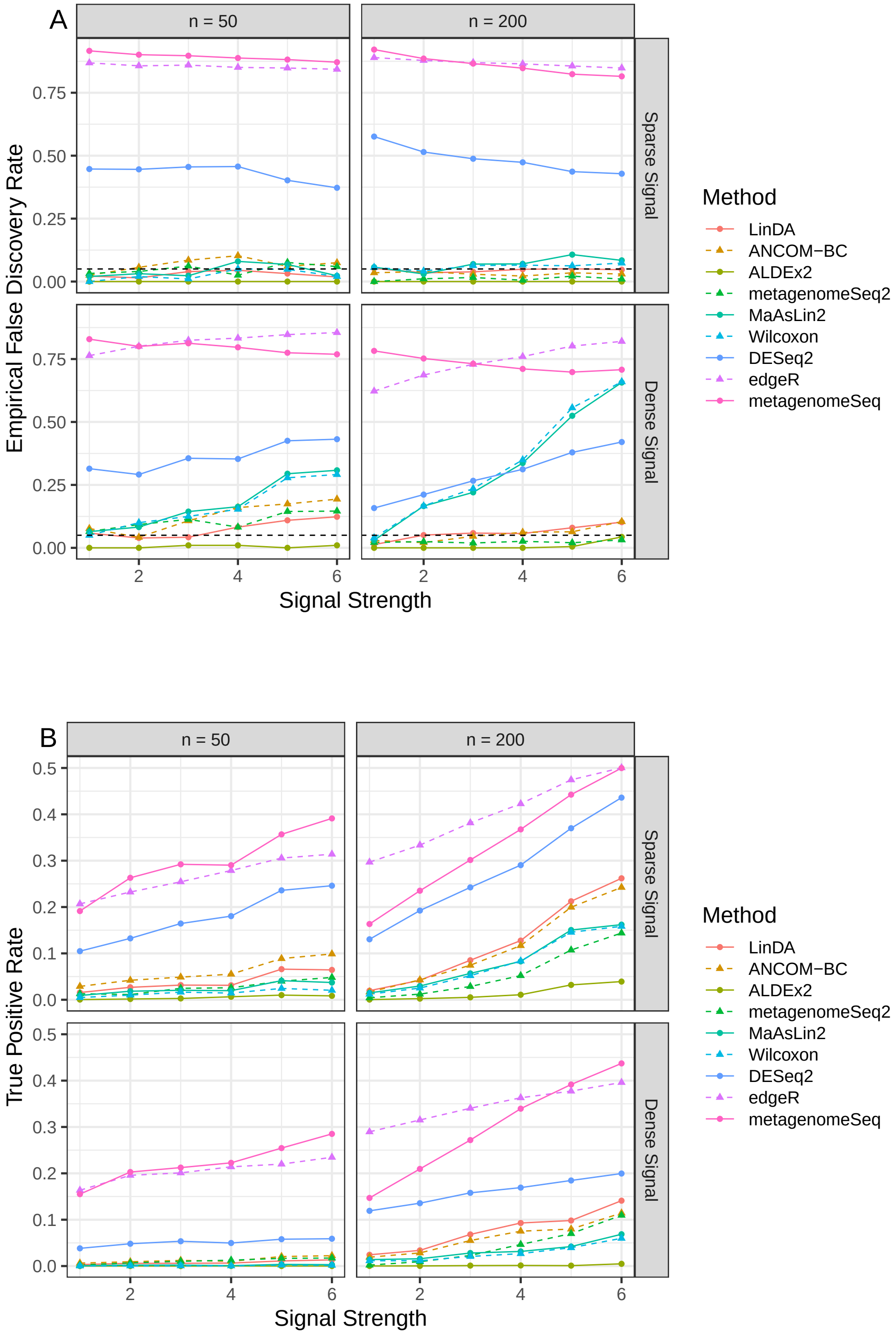

Fig. S30: Full performance comparison (S0C0 with strong compositional effects). Empirical false discovery rate (A) and true positive rates (B) were averaged over 100 simulation runs. The dashed horizontal line (A) indicates the target FDR level of 0.05.
